# Secular Trend in Glycaemic Management in Type 2 Diabetes Patients With and Without Cirrhosis Between 2000 and 2023: A Territory‐Wide Cohort Study

**DOI:** 10.1111/apt.70705

**Published:** 2026-05-05

**Authors:** Mary Yue Wang, Sherlot Juan Song, Nana Peng, Grace Lai‐Hung Wong, Vincent Wai‐Sun Wong, Jimmy Che‐To Lai, Terry Cheuk‐Fung Yip

**Affiliations:** ^1^ Medical Data Analytics Centre The Chinese University of Hong Kong Shatin Hong Kong SAR China; ^2^ Department of Medicine and Therapeutics The Chinese University of Hong Kong Shatin Hong Kong SAR China; ^3^ State Key Laboratory of Digestive Disease The Chinese University of Hong Kong Shatin Hong Kong SAR China; ^4^ Li Ka Shing Institute of Health Sciences The Chinese University of Hong Kong Shatin Hong Kong SAR China

**Keywords:** cirrhosis, glycaemic control, hypoglycaemia, treatment, type 2 diabetes mellitus

## Abstract

**Background and Aims:**

With the change in antidiabetic medications, the trend of glycaemic management is still unclear for type 2 diabetes mellitus (T2DM) patients, especially those with cirrhosis. We aimed to examine secular trends in glycaemic control and antidiabetic medication usage among T2DM patients with or without cirrhosis.

**Methods:**

We retrospectively included T2DM adult patients with or without cirrhosis from 2000 to 2023 in Hong Kong, and excluded those with type 1 diabetes. Serial laboratory measurements in each of the five consecutive periods (2000–2004, 2005–2009, 2010–2014, 2015–2019, and 2020–2023) were compared by time‐weighted average. The percentages of patients who achieved haemoglobin A_1c_ (HbA_1c_) < 7% and fasting blood glucose < 7.2 mmol/L, as well as the trend of antidiabetic medication usage and hypoglycaemia incidence, were examined.

**Results:**

Of 1,206,233 patients with T2DM (mean age 63.1 years, 52.0% males), 1,143,033 (94.8%), 45,208 (3.7%) and 17,992 (1.5%) had no cirrhosis, compensated and decompensated cirrhosis, respectively. All patients with or without cirrhosis had increasing proportions of patients achieving HbA_1c_ target over the years, with compensated cirrhosis group having the best control (from 2000–04 to 2020–23: non‐cirrhosis group: 39.2% to 65.9%, compensated cirrhosis group: 49.3% to 75.8%, decompensated cirrhosis group: 47.6% to 64.6%). The probability of achieving glycaemic targets increased over time in all groups. Insulin was the predominant agent in decompensated cirrhosis group, with an increasing incidence of hypoglycaemia.

**Conclusion:**

Glycaemic control improved in T2DM patients irrespective of cirrhosis status. Further research is needed to determine the impact of sustained control on hepatic and extrahepatic outcomes.

## Graphical abstract

In this territory‐wide cohort study, improving glycemic control can be observed regardless of cirrhosis status. Oral antidiabetic medication was more often used in the non‐cirrhosis group, and insulin was preferred in patients with decompensated cirrhosis, who exhibited an increasing incidence of hypoglycemia over the years and need individualized blood glucose management.

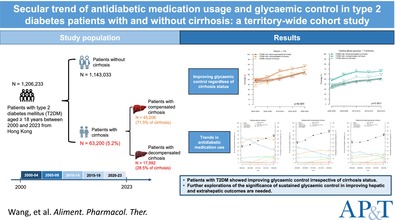

## 1 Introduction

Type 2 diabetes mellitus (T2DM) is a significant public health challenge, currently affecting over 536.6 million individuals worldwide and expected to affect 783.2 million people in 2045, posing threats to global health [1]. The relationship between T2DM and liver disease, especially metabolic dysfunction‐associated steatotic liver disease (MASLD), is well known, exacerbating each other by sharing pathways like insulin resistance and dysbiosis [2]. Global prevalence of MASLD in patients with T2DM has risen from 55.9% in 1990–2004 to 68.8% in 2016–2021 [3]. T2DM patients have a higher chance of developing advanced liver diseases and cirrhosis, leading to worse clinical outcomes [4, 5]. As an important monitoring parameter, elevated haemoglobin A_1c_ (HbA_1c_) is also an independent contributor to progression to hepatic decompensation and hepatocellular carcinoma (HCC) [6].

Over the past decades, sodium‐glucose co‐transporter‐2 inhibitors (SGLT‐2is) and glucagon‐like peptide‐1 receptor agonists (GLP‐1 RAs) and other new antidiabetic treatments have revolutionised T2DM management [7]. However, glycaemic control in patients with cirrhosis is still challenging due to the risk of hypoglycaemia, specialised nutritional requirements, renal impairment, and medication‐related side effects such as metformin‐associated lactic acidosis [8, 9]. Insulin is the preferred option in glycaemic control of patients with cirrhosis, especially for those with decompensated cirrhosis, while close glucose monitoring is needed [7]. In the latest American Diabetes Association (ADA) guidelines, HbA_1c_ level < 7% and fasting blood glucose (FBG) < 7.2 mmol/L are the recommended targets to reduce the risk of hepatic and extrahepatic complications in individuals with T2DM [10]. Nonetheless, the trend of glycaemic control and the impact of changing medication patterns in patients with T2DM and cirrhosis remain unclear. Therefore, this study aimed to compare the secular trends in glycaemic control and antidiabetic medication usage among individuals with T2DM, with and without cirrhosis.

## 2 Materials and Methods

### 2.1 Study Design and Data Source

This was a territory‐wide, retrospective cohort study in Hong Kong using data from the Clinical Data Analyses and Reporting System (eMethods). The study was from January 1, 2000 to December 31, 2023, separated into five consecutive intervals: 2000–2004, 2005–2009, 2010–2014, 2015–2019, and 2020–2023. This study followed the guidelines of Strengthening the Reporting of Observational Studies in Epidemiology. Ethical approval for this study was obtained from the Joint Chinese University of Hong Kong–New Territories East Cluster Clinical Research Ethics Committee (Reference Number: 2022.064). Informed consent was waived due to the retrospective nature.

### 2.2 Participants

**FIGURE 1:**
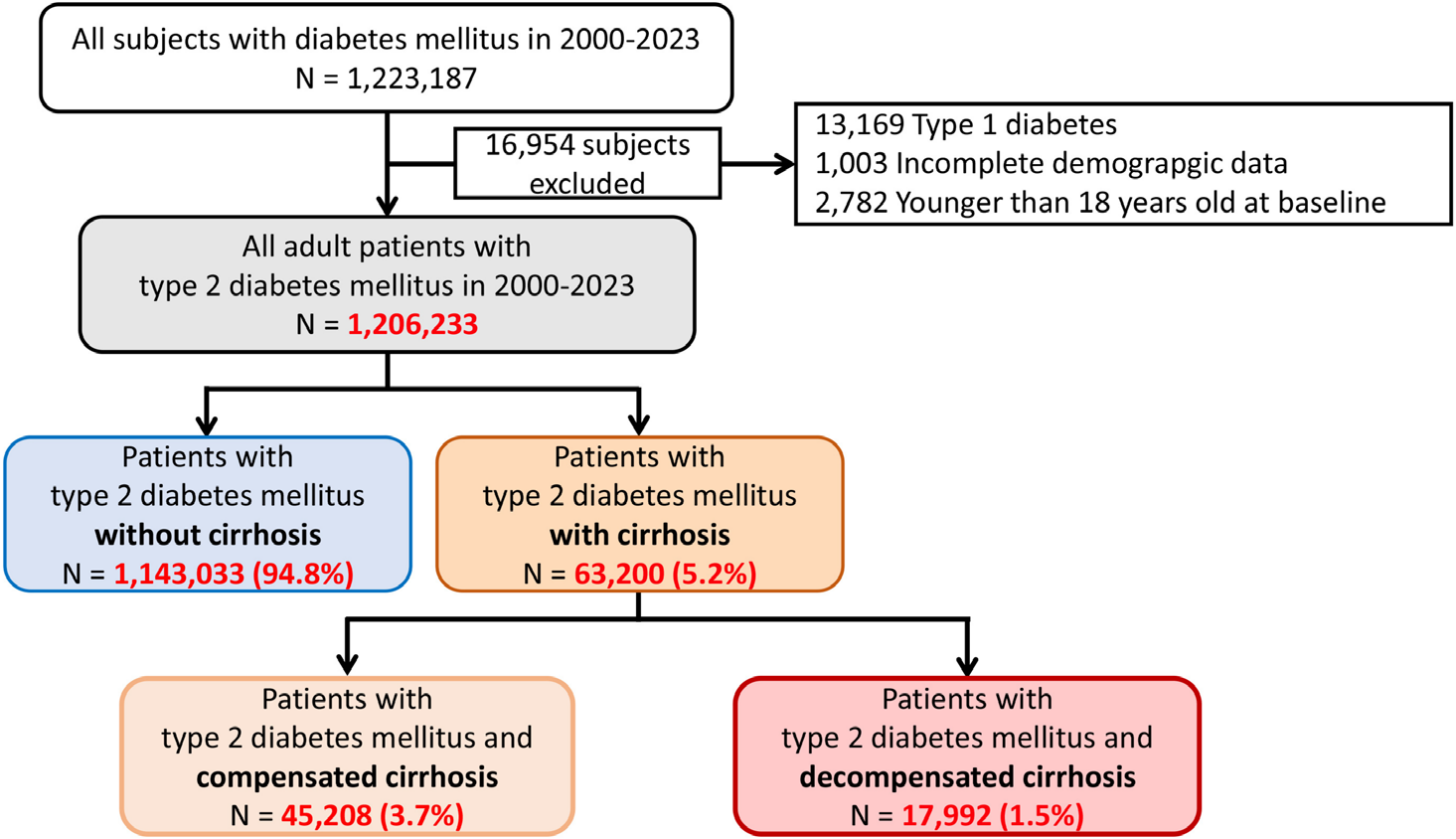
Flowchart of participant selection.

Patients with T2DM who had at least one record of HbA_1c_ and fasting blood glucose were identified if they met at least one of the following criteria: (1) International Classification of Diseases (ICD) diagnoses of T2DM; (2) continuous use of insulin for at least 28 days; (3) any use of antidiabetic drugs other than insulin; (4) HbA_1c_ ≥ 6.5% (equivalent to 48 mmol/mol); and/or (5) FBG ≥ 7.0 mmol/L. The date of T2DM diagnosis was defined as the first date of occurrence of any of the above criteria for T2DM. Patients with gestational diabetes were not included in our study (ICD‐9 codes: 648.8). Patients were excluded if they: (1) had incomplete demographic data; (2) aged < 18 years at T2DM diagnosis; and/or (3) were diagnosed as type 1 diabetes mellitus (Figure 1).

### 2.3 Data Collection

Demographic data (date of birth and gender), anthropometric data, and laboratory data (alanine aminotransferase [ALT], aspartate aminotransferase [AST], albumin, total bilirubin, creatinine, platelet counts, and lipid profiles) were captured and compared between groups with and without cirrhosis. Diagnosis and procedure codes on comorbidities and data on antidiabetic or other medication records were also collected (Table S1).

### 2.4 Definitions

Patients fulfilling any of the following criteria were identified as having cirrhosis: (1) diagnosis codes of cirrhosis; (2) diagnosis codes related to decompensated events (ascites, variceal bleeding, hepatic encephalopathy, hepatorenal syndrome, spontaneous bacterial peritonitis, or hepatopulmonary syndrome), listed in Table S1 (defined as decompensated cirrhosis); (3) the presence of portal hypertension or gastric/oesophageal varices; (4) fibrosis‐4 (FIB‐4) index > 3.25, and/or an AST to platelet ratio index (APRI) > 2 as previously described [11]. To stratify different stages of cirrhosis, decompensated cirrhosis was defined by any presence of hepatic decompensation, and compensated cirrhosis was identified without any record of the events. The date of cirrhosis diagnosis was defined as the earliest date of occurrence of any criteria for cirrhosis. Definitions of comorbidities were described in eMethods.

### 2.5 Primary and Secondary Outcomes

The primary outcome was the achievement of glycaemic target defined as HbA_1c_ < 7%, while the secondary outcome was the achievement of FBG < 7.2 mmol/L, following ADA guideline recommendation [12]. Only patients with at least one record of HbA_1c_ and fasting blood glucose in each interval were included in the analysis. Multiple HbA_1c_ and FBG measurements for each patient across a period were summarised by time‐weighted average (TWA). Percentages of patients meeting the glycaemic targets among those without cirrhosis, with compensated cirrhosis, and decompensated cirrhosis were compared across five consecutive periods. Each period consisted of newly diagnosed patients with T2DM in that period and prevalent T2DM in previous periods. The follow‐up started at the date of T2DM diagnosis for patients without cirrhosis, and for those with cirrhosis, the follow‐up started at the latest date between T2DM and cirrhosis diagnoses. To mitigate the risk of misinterpretation of data due to extreme measurements at initial diagnosis, the percentage was calculated using records one year post‐diagnosis. Considering the small changes across the target may represent only modest clinical improvement, proportions of patients reaching HbA_1c_ < 6.5% and < 8% were also analysed. Sensitivity analyses were conducted, limited to newly diagnosed T2DM cases in one period, and another sensitivity analysis for all available glucose records post‐T2DM and cirrhosis diagnoses.

Other secondary outcomes were changes in medication utilization patterns among patients with or without cirrhosis. The internal drug codes of the Hospital Authority were employed to identify relevant medications. The use of antidiabetic medications (metformin, sulfonylurea, insulin, thiazolidinedione [TZD], α‐glucosidase inhibitor, dipeptidyl peptidase‐4 inhibitor [DPP‐4i], SGLT‐2i and GLP‐1 RA), anti‐hypertensive medications (angiotensin‐converting enzyme inhibitor, angiotensin receptor blocker, beta blocker, calcium channel blocker, and thiazide diuretic) and lipid‐lowering agents (statin and other lipid‐lowering agents) were examined. Medication exposure was defined as at least one prescription record for the drug of interest within the period. If the medication was a combination product, each component was counted as a distinct agent in the period. Other concomitant medications included angiotensin‐converting enzyme inhibitors, angiotensin receptor blockers, beta blockers, calcium channel blockers, thiazide diuretics, statin, other lipid‐lowering agents, and aspirin. Medication records from 90 days post‐diagnosis of T2DM and cirrhosis were analysed for prescription changes. Logistic regression was conducted to examine the possible impact on the likelihood of achieving HbA_1c_ target. A sensitivity analysis was conducted on medication use from 30 days post‐diagnosis. The trend of hypoglycaemia incidence was also compared between groups.

### 2.6 Statistical Analyses

Continuous variables were expressed as mean ± standard deviation (SD) or median (IQR), and categorical variables were presented as numbers. Group differences across two periods were analysed using chi‐square or Fisher's exact tests for categorical data and ANCOVA for comparing changes among patient groups. Linear mixed model and mixed effects logistic regression were used to assess the quantitative and qualitative differences in trend across the five consecutive periods among different groups. Another mixed effects logistic regression model was built to quantify the period effect on achieving glycaemic target. Univariate and multivariable analyses were performed to estimate the odds ratio (OR) and adjusted OR (aOR) for target achievement, considering potential confounding factors. The following factors are considered: age at the beginning of each period; gender; body mass index; hypertension; dyslipidaemia; hepatitis virus infection; excessive alcohol intake; laboratory measurements (including estimated glomerular filtration rate, total bilirubin, total cholesterol, platelet count, albumin, ALT and AST); the usage of antidiabetic medication (sulfonylureas, metformin, thiazolidinedione, SGLT‐2i, GLP‐1 RA, DPP‐4i, α‐glucosidase inhibitors and insulin); angiotensin converting enzyme inhibitors (ACEI); angiotensin receptor blockers (ARB); beta blockers; calcium channel blockers; thiazide diuretics; statin; lipid‐lowering agents other than statin; and aspirin. For primary outcome, subgroup analysis stratifying patients by age (< 65 years and ≥ 65 years) and key comorbidities (hypertension, chronic kidney disease and major adverse cardiovascular events) was conducted. Missing data were regarded as missing at random and replaced with substituted data from five complete data sets by chained equations. All statistical tests were two‐sided, and *p*‐value < 0.05 was taken as statistically significant. Statistical analyses were performed using R software (4.3.1; R Foundation for Statistical Computing).

## 3 Results

### 3.1 Patients' Characteristics

1,206,233 patients with T2DM were included, with 63,200 (5.2%) having cirrhosis. 17,992 (1.5%) had decompensated cirrhosis, and 45,208 (3.7%) had compensated cirrhosis (Figure 1). More patients with cirrhosis were male (decompensated cirrhosis vs. compensated cirrhosis vs. non‐cirrhosis in 2020–2023: 61.3% vs. 60.1% vs. 51.4%, *p* < 0.001), and those with decompensated cirrhosis had the highest proportion of hepatitis virus infection (decompensated cirrhosis vs. compensated cirrhosis vs. non‐cirrhosis in 2020–2023: 40.0% vs. 25.3% vs. 6.2%, *p* < 0.001) and excessive alcohol use (decompensated cirrhosis vs. compensated cirrhosis vs. non‐cirrhosis in 2020–2023: 34.4% vs. 12.8% vs. 1.2%, *p* < 0.001) (Table 1).

**TABLE 1:** Basic clinical characteristics of patients with type 2 diabetes mellitus without cirrhosis, with compensated cirrhosis, and with decompensated cirrhosis.

Characteristics	Patients without cirrhosis^a^					Patients with compensated cirrhosis^b^					Patients with decompensated cirrhosis^c^					*p*
	2000–04 *N* = 274,527	2005–09 *N* = 414,676	2010–14 *N* = 587,307	2015–19 *N* = 765,409	2020–23 *N* = 900,553	2000–04 *N* = 7917	2005–09 *N* = 11,485	2010–14 *N* = 16,756	2015–19 *N* = 20,985	2020–23 *N* = 25,719	2000–04 *N* = 2249	2005–09 *N* = 3800	2010–14 *N* = 5792	2015–19 *N* = 6898	2020–23 *N* = 6650	
Baseline age (years)	64.2 ± 13.1	62.5 ± 13.3	61.4 ± 13.0	60.7 ± 12.9	60.2 ± 12.7	70.0 ± 12.7	69.1 ± 13.5	69.0 ± 14.0	67.9 ± 14.1	67.6 ± 14.2	63.5 ± 11.2	62.5 ± 11.6	62.2 ± 11.7	61.7 ± 11.9	60.7 ± 11.9	< 0.001
Male gender (*n*, %)	125,236 (46.7)	199,136 (48.9)	290,377 (50.0)	387,636 (50.9)	462,884 (51.4)	5487 (56.9)	7913 (58.4)	11,056 (59.0)	13,392 (59.9)	15,503 (60.1)	3772 (54.8)	5220 (57.0)	6138 (59.2)	5660 (60.4)	4031 (61.3)	< 0.001
BMI (kg/m^2^)	25.4 ± 6.6	25.6 ± 4.3	25.8 ± 4.2	25.7 ± 4.6	25.6 ± 4.6	24.7 ± 3.6	24.9 ± 3.9	25.1 ± 4.7	24.3 ± 4.8	23.8 ± 5.1	24.8 ± 3.7	25.2 ± 4.0	25.1 ± 4.3	24.5 ± 4.9	24.3 ± 4.7	< 0.001
Missing (%)	85.2	69.5	46.4	36.8	44.4	93.9	86.6	74.6	53.7	48.5	92.4	81.6	67.6	44.7	38.6	—
Overweight (*n*, %)	9219 (22.7)	28,619 (22.6)	67,694 (21.5)	99,598 (20.6)	98,981 (19.8)	110 (22.9)	355 (23.1)	885 (20.8)	1932 (19.9)	2452 (18.5)	40 (23.4)	146 (20.8)	389 (20.7)	744 (19.5)	762 (18.7)	< 0.001
Obesity (*n*, %)	20,466 (50.3)	64,816 (51.2)	168,591 (53.6)	255,597 (52.8)	255,001 (50.9)	211 (43.9)	700 (45.6)	2014 (47.3)	3822 (39.3)	4707 (35.6)	75 (43.9)	343 (48.9)	878 (46.7)	1518 (39.8)	1586 (38.9)	< 0.001
Hypertension (*n*, %)	186,794 (68.0)	308,573 (74.4)	462,547 (78.8)	604,279 (78.9)	692,929 (76.9)	5716 (72.2)	8691 (75.7)	13,521 (80.7)	16,937 (80.7)	20,350 (79.1)	1762 (78.3)	3151 (82.9)	5015 (86.6)	5889 (85.4)	5536 (83.2)	< 0.001
Dyslipidaemia (*n*, %)	115,922 (42.2)	204,965 (49.4)	397,586 (67.7)	395,049 (51.6)	405,914 (45.1)	2867 (36.2)	5198 (45.3)	8871 (52.9)	8392 (40.0)	9460 (36.8)	506 (22.5)	1348 (35.5)	2249 (38.8)	2702 (39.2)	2398 (36.1)	< 0.001
CKD (*n*, %)	72,816 (35.1)	101,261 (30.6)	131,894 (24.7)	165,264 (23.8)	186,616 (23.3)	4106 (55.9)	5071 (47.5)	6775 (43.4)	7918 (41.1)	8799 (37.8)	1127 (59.9)	2141 (59.9)	3161 (57.8)	3632 (56.6)	3406 (56.6)	< 0.001
Chronic viral hepatitis (*n*, %)	5195 (1.9)	11,943 (2.9)	22,287 (3.8)	40,100 (5.2)	56,212 (6.2)	880 (11.1)	1712 (14.9)	3160 (18.9)	4796 (22.9)	6504 (25.3)	934 (41.5)	1714 (45.1)	2546 (44.0)	2744 (39.8)	2660 (40.0)	< 0.001
Excessive alcohol use (*n*, %)	987 (0.4)	2375 (0.6)	4541 (0.8)	8142 (1.1)	10,562 (1.2)	555 (7.0)	1150 (10.0)	1995 (11.9)	2775 (13.2)	3280 (12.8)	836 (37.2)	1641 (43.2)	2339 (40.1)	2489 (36.1)	2287 (34.4)	< 0.001
TWA HbA_1c_ (%)	7.6 ± 1.5	7.4 ± 1.4	7.1 ± 1.2	7.0 ± 1.1	6.9 ± 1.0	7.3 ± 1.7	7.1 ± 1.5	6.8 ± 1.3	6.6 ± 1.2	6.5 ± 1.1	7.6 ± 1.6	7.2 ± 1.6	7.1 ± 1.4	6.8 ± 1.4	6.6 ± 1.3	< 0.001
TWA FBG (mmol/L)	8.1 ± 2.5	7.7 ± 2.2	7.3 ± 2.0	7.3 ± 1.9	7.1 ± 1.8	8.0 ± 2.8	7.3 ± 2.4	7.0 ± 2.2	7.0 ± 2.0	6.9 ± 2.1	8.5 ± 3.3	7.9 ± 2.8	7.6 ± 2.6	7.5 ± 2.5	7.2 ± 2.3	< 0.001
Patients without HbA_1c_ or FBG (%)	38.6	34.7	14.1	13.1	17.2	37.9	37.4	24.2	19.1	21.8	49.0	24.3	20.5	18.5	24.7	

*Note:* In each interval, only patients with both HbA_1c_ and fasting blood glucose records were included for glycaemic control analysis, excluding those without any HbA_1c_ or fasting blood glucose. To assess group differences across two periods, chi‐square or Fisher's exact tests for categorical data and ANCOVA for comparing changes among patient groups were used. Linear mixed model and mixed effects logistic regression were used to assess the quantitative and qualitative differences in trend across the five consecutive periods among different groups.

Abbreviations: BMI, body mass index; CKD, chronic kidney disease; FBG, fasting blood glucose; HbA_1c_, haemoglobin A_1c_; T2DM, type 2 diabetes mellitus; TWA, Time‐weighted average.

^a^ All *p*‐values < 0.001 for linear trend in consecutive periods in patients without cirrhosis.

^b^ All *p*‐values < 0.001 for linear trend in consecutive periods in patients with compensated cirrhosis.

^c^ All *p*‐values < 0.001 for linear trend in consecutive periods in patients with decompensated cirrhosis.

### 3.2 Secular Trend of HbA_1c_ and Other Laboratory Measurements

**FIGURE 2:**
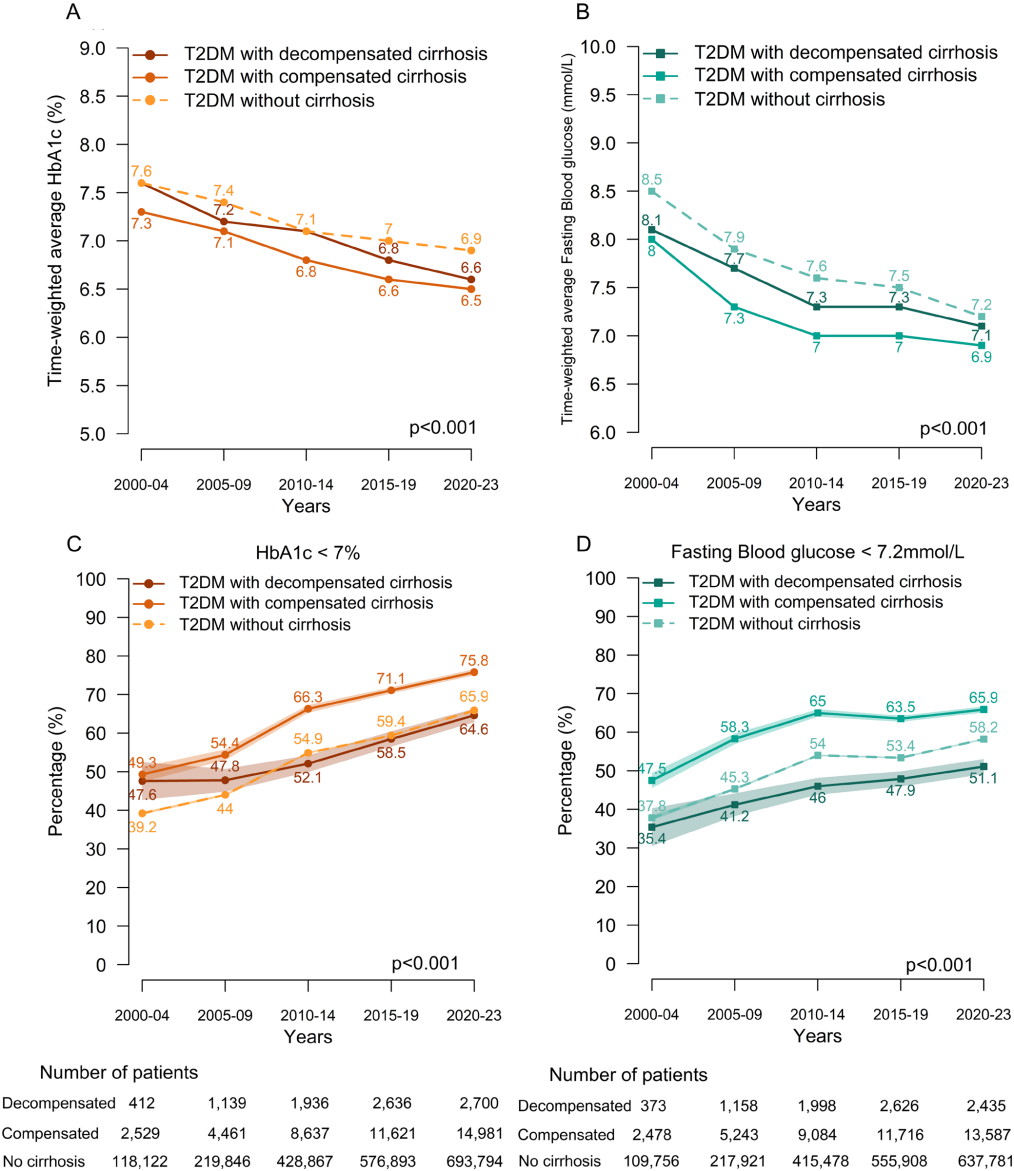
Secular trend of (A) haemoglobin A_1c_ and (B) fasting blood glucose and proportions of patients achieving (C) haemoglobin A_1c_ and (D) fasting blood glucose targets among patients with type 2 diabetes mellitus who did not have cirrhosis, had compensated cirrhosis, and had decompensated cirrhosis. Abbreviations: HbA_1c_, haemoglobin A_1c_. To assess the quantitative and qualitative differences in trend across the five consecutive periods among different groups, linear mixed model and mixed effects logistic regression were conducted.

Patients with compensated cirrhosis had the most frequent measurement per year, with the median number of 1.46 (0.80, 2.30) of HbA_1c_ and 1.36 (0.76, 2.38) of FBG, compared to 1.38 (0.81, 1.97) measurements of HbA_1c_ and 1.10 (0.70, 1.66) measurements of FBG in the non‐cirrhosis group, and 1.43 (0.84, 2.14) measurements of HbA_1c_ and 1.30 (0.76, 2.14) measurements of FBG for those with decompensated cirrhosis (Table S3). Over the five consecutive periods, TWA HbA_1c_ of patients without cirrhosis decreased from 7.6% to 6.9%, while patients with compensated and decompensated cirrhosis decreased from 7.3% to 6.5%, and from 7.6% to 6.6%, respectively (*p* < 0.001 for both differences in overall mean and linear trend). Similarly, TWA FBG levels decreased from 8.5 mmol/L to 7.2 mmol/L in patients without cirrhosis, 8.0 mmol/L to 6.9 mmol/L for those with compensated cirrhosis, and 8.1 mmol/L to 7.1 mmol/L in the decompensated cirrhosis group (*p* < 0.001 for both differences in overall mean and linear trend) (Figures 2A,B and Table 1). Both compensated and decompensated cirrhosis groups consistently showed elevated liver enzymes and creatinine with lower platelets and albumin than the non‐cirrhosis group (Table S4).

### 3.3 Proportions of Patients Who Achieved Glycaemic Targets

The proportion of patients meeting the HbA_1c_ target was consistently increasing in both cirrhosis and non‐cirrhosis groups (Figure 2C). Specifically, the proportion of individuals achieving the HbA_1c_ target increased from 49.3% (95% confidence interval [CI], 48.5%–51.1%) to 75.8% (95% CI, 75.2%–76.3%) among patients with compensated cirrhosis, from 47.6% (95% CI, 44.7%–50.9%) to 64.6% (95% CI, 61.6%–65.9%) in patients with decompensated cirrhosis, and 39.2% (95% CI, 38.8%–39.6%) to 65.9% (95% CI, 65.5%–66.7%) in patients without cirrhosis (*p* < 0.001 for both differences in overall mean and linear trend). A similar increasing trend in the proportion of patients achieving the FBG target of < 7.2 mmol/L was also observed (Figure 2D). Patients with compensated cirrhosis demonstrated better FBG control, with the proportion of individuals achieving the target increasing from 47.5% (95% CI, 45.9%–49.5%) to 65.9% (95% CI, 64.8%–66.1%), compared to the improvement from 37.8% (95% CI, 37.2%–38.4%) to 58.2% (95% CI, 57.7%–58.9%) in the non‐cirrhosis group. The decompensated cirrhosis group exhibited the lowest proportion of achieving the target, improving from 35.4% (95% CI, 31.4%–40.2%) to 51.1% (95% CI, 49.5%–52.3%) (*p* < 0.001 for both differences in overall mean and linear trend). When analysing proportions of patients reaching HbA_1c_ < 6.5% and < 8%, similar improvement can still be observed in all groups (Table S5). In sensitivity analyses, we incorporated all available HbA_1c_ and FBG measurements in the follow‐up period (Figure S1A), and one only included patients newly diagnosed with T2DM in each period, capturing all their measurements (Figure S1B), as well as another one considering only the glycaemic records 1 year after diagnosis (Figure S1C). The improving trends remained consistent with the primary results. The improving trend in HbA_1c_ control was consistently observed across all subgroups stratified by age, hypertension, CKD and MACE (all *p* for linear trend < 0.001, Table S8).

### 3.4 Trends in Medication Patterns

**FIGURE 3:**
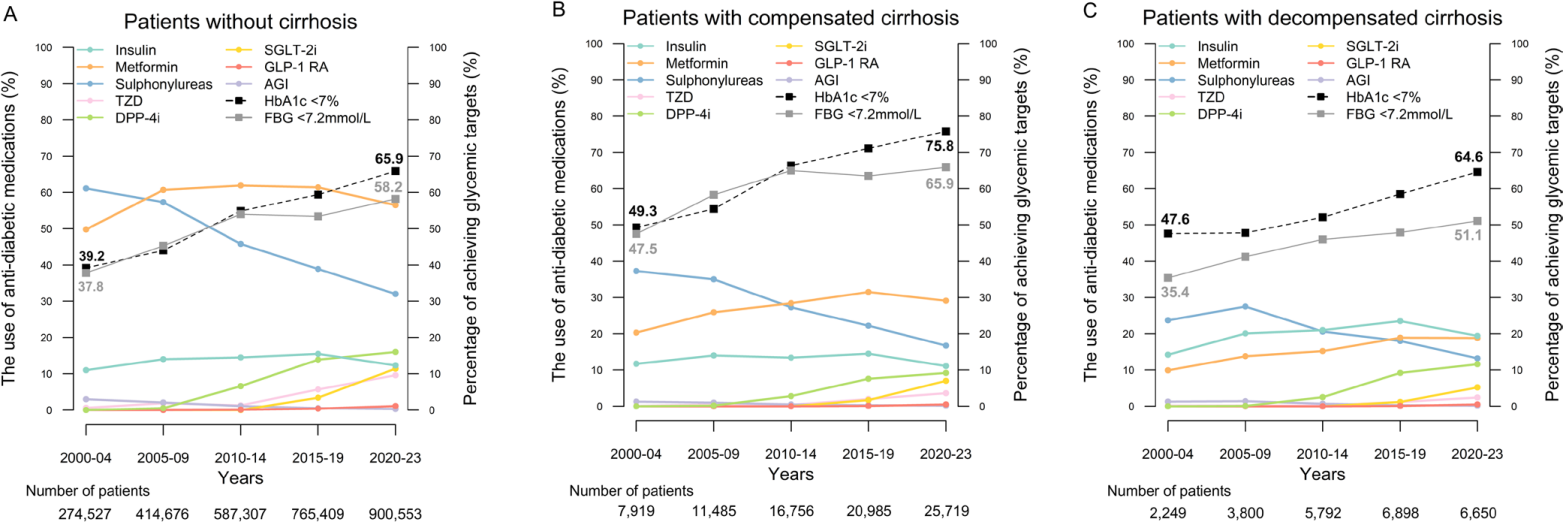
Trends of medication patterns and haemoglobin A_1c_, fasting blood glucose among (A) patients without cirrhosis, (B) with compensated cirrhosis, and (C) decompensated cirrhosis. Abbreviations: TZD, thiazolidinedione; SGLT‐2i, sodium‐glucose cotransporter 2 inhibitor; GLP‐1A, glucose like peptide‐1 receptor agonists; DPP‐4i, dipeptidyl peptidase‐4 inhibitor; AGI, Acarbose. HbA_1c_, haemoglobin A_1c_. FBG, fasting blood glucose.

The median duration and dose of each kind of anti‐diabetic medications was summarised in Table S2. Sulfonylureas, metformin, and insulin were the most commonly prescribed medications in all groups (Figure 3). The utilisation of sulfonylureas declined consistently in all groups, whereas the use of metformin remained stable (Figure S2A,B). There has been an increase in the utilisation of TZD, DPP‐4i, SGLT‐2i and GLP‐1 RA in recent years (Figure S2C–F). Overall, patients with cirrhosis received fewer oral medications than those without cirrhosis (Figure 3). More patients without cirrhosis were prescribed metformin than patients with compensated and decompensated cirrhosis (56.5% vs. 29.1% vs. 18.8%, respectively in 2020–2023, *p* < 0.001). More patients with decompensated cirrhosis were prescribed insulin, with its utilisation rate increasing from 14.2% to 19.4%, compared to stable trends in patients with compensated cirrhosis (from 11.7% to 11.1%) and without cirrhosis (from 11.0% to 12.3%). When investigating medication records 30 days post‐diagnosis of cirrhosis, the results remained consistent with the primary findings (Figure S3).

### 3.5 Year Period, Cirrhosis Status and the Likelihood of Achieving Glycaemic Targets

Following adjustments for confounding factors including demographics, comorbidities, excessive alcohol usage, laboratory tests, and antidiabetic medication use, the proportion of patients meeting glycaemic targets remained improving over the years among individuals with or without cirrhosis. Compared to patients without cirrhosis, patients with compensated cirrhosis were more likely to achieve HbA_1c_ target (aOR 1.41 [95% CI, 1.37–1.45]), while patients with decompensated cirrhosis were less likely to achieve HbA_1c_ target (aOR 0.88 [95% CI, 0.84–0.92]). Similar findings were observed in analyses of FBG (aOR 1.08 [95% CI, 1.06–1.11] in compensated cirrhosis; aOR 0.87 [95% CI, 0.84–0.91] in decompensated cirrhosis) (Table 2). In multivariable analyses adjusting for cirrhosis status and other covariates, the use of most antidiabetic medications was associated with a lower likelihood of achieving glycaemic targets compared with non‐users (Tables S6 and S7), indicating patients with poorer glycaemic control were more likely to receive these agents.

**TABLE 2:** Association between the year period and the likelihood of achieving glycaemic targets among patients without cirrhosis, with compensated cirrhosis, and decompensated cirrhosis.

Group	Odds ratio (95% confidence interval) of achieving HbA_1c_ target		Odds ratio (95% confidence interval) of achieving FBG target	
	Univariate analysis	Multivariable analysis	Univariate analysis	Multivariable analysis
No cirrhosis				
2000–2004	1 [Reference]	1 [Reference]	1 [Reference]	1 [Reference]
2005–2009	1.19 (1.17, 1.21)	1.82 (1.79, 1.85)	1.49 (1.47, 1.52)	1.89 (1.86, 1.91)
2010–2014	1.68 (1.65, 1.70)	2.63 (2.58, 2.67)	2.21 (2.18, 2.24)	2.81 (2.77, 2.85)
2015–2019	1.95 (1.92, 1.98)	3.10 (3.05, 3.15)	2.18 (2.15, 2.21)	2.69 (2.65, 2.73)
2020–2023	2.47 (2.44, 2.51)	4.18 (4.11, 4.24)	2.69 (2.66, 2.73)	3.41 (3.36, 3.46)
Compensated cirrhosis				
2000–2004	1 [Reference]	1 [Reference]	1 [Reference]	1 [Reference]
2005–2009	1.42 (1.30, 1.55)	1.94 (1.77, 2.12)	1.61 (1.49, 1.73)	1.70 (1.57, 1.84)
2010–2014	2.33 (2.14, 2.54)	3.30 (3.02, 3.61)	2.34 (2.18, 2.51)	2.49 (2.31, 2.69)
2015–2019	2.88 (2.65, 3.13)	4.26 (3.89, 4.66)	2.42 (2.25, 2.60)	2.58 (2.39, 2.79)
2020–2023	3.56 (3.27, 3.87)	5.58 (5.09, 6.11)	2.80 (2.61, 3.00)	3.02 (2.80, 3.26)
Decompensated cirrhosis				
2000–2004	1 [Reference]	1 [Reference]	1 [Reference]	1 [Reference]
2005–2009	1.06 (0.92, 1.23)	1.66 (1.43, 1.93)	1.36 (1.16, 1.58)^a^	1.62 (1.38, 1.90)
2010–2014	1.28 (1.12, 1.47)	2.13 (1.84, 2.47)	1.69 (1.46, 1.96)	2.05 (1.76, 2.39)
2015–2019	1.74 (1.52, 1.97)	2.93 (2.53, 3.40)	1.85 (1.60, 2.14)	2.19 (1.87, 2.55)
2020–2023	2.24 (1.96, 2.56)	3.91 (3.36, 4.56)	2.13 (1.84, 2.46)	2.51 (2.15, 2.94)
*p*‐value comparing trend of no cirrhosis, compensated cirrhosis, and decompensated cirrhosis	0.119	0.747	0.838	0.270
All patients				
2000–2004	1 [Reference]	1 [Reference]	1 [Reference]	1 [Reference]
2005–2009	1.19 (1.18, 1.21)	1.83 (1.80, 1.86)	1.50 (1.48, 1.52)	1.88 (1.85, 1.90)
2010–2014	1.69 (1.66, 1.71)	2.64 (2.60, 2.69)	2.21 (2.18, 2.24)	2.79 (2.76, 2.83)
2015–2019	1.96 (1.94, 1.99)	3.13 (3.08, 3.18)	2.18 (2.15, 2.21)	2.69 (2.65, 2.73)
2020–2023	2.49 (2.46, 2.52)	4.22 (4.15, 4.29)	2.69 (2.66, 2.72)	3.40 (3.35, 3.45)
All patients				
No cirrhosis	1 [Reference]	1 [Reference]	1 [Reference]	1 [Reference]
Compensated cirrhosis	1.76 (1.71–1.82)	1.41 (1.37–1.45)	1.16 (1.14, 1.19)	1.08 (1.06, 1.11)
Decompensated cirrhosis	0.98 (0.94–1.03)	0.88 (0.84–0.92)	0.81 (0.78, 0.85)	0.87 (0.84, 0.91)

*Note:* Multivariable analysis: Adjustment for age, gender, body mass index, type 2 diabetes mellitus duration, hypertension, dyslipidaemia, hepatitis virus infection, excessive alcohol intake, estimated glomerular filtration rate (eGFR), total bilirubin, total cholesterol, platelet, albumin, alanine aminotransferase, aspartate aminotransferase and medication use. Cirrhosis status was adjusted for all patients.

Abbreviations: FBG, fasting blood glucose; HbA_1c_, haemoglobin A_1c_.

^a^ *p*‐value: 0.009, all other *p*‐values < 0.001 versus reference.

### 3.6 Trends in Hypoglycaemia Rate

There were decreasing trends in the incidence of hypoglycaemia over years in patients without cirrhosis and with compensated cirrhosis (per 100 person‐years, from 2000–2004 to 2020–2023, 1.73 (95% CI, 1.70–1.76) to 0.36 (95% CI, 0.34–0.38) in the non‐cirrhosis group, and 3.64 (95% CI, 3.34–3.96) to 2.25 (95% CI, 2.02–2.49) in the compensated cirrhosis group), while the incidence increased in the decompensated cirrhosis group, from 9.62 (95% CI, 8.38–11.0) to 20.4 (95% CI, 18.7–22.2) per 100 person‐years over years (Table S9).

## 4 Discussion

While there has been improving glycaemic control in patients with T2DM over the past two decades, whether the same is true in patients with cirrhosis remained unknown. This retrospective cohort study revealed that patients with compensated cirrhosis showed a greater improvement in glycaemic levels compared to those without cirrhosis, whereas individuals with decompensated cirrhosis experienced less improvement in glycaemic control over time. Oral antidiabetic medications, particularly metformin, were more commonly used in patients without cirrhosis, while insulin predominated the treatment in individuals with cirrhosis, especially decompensated cirrhosis, potentially leading to an increase in hypoglycaemia incidence in these patients.

The improvement in glycaemic control aligned with previous studies, which showed an overall improving or stable trend of HbA_1c_ levels in T2DM population [13, 14]. Considering the need for cautious interpretation of HbA_1c_ due to lower haemoglobin levels from hypersplenism in patients with cirrhosis, our analyses of FBG further supported the primary findings. Considering the need for cautious interpretation of HbA_1c_ due to lower haemoglobin levels from hypersplenism in patients with cirrhosis, the FBG level provides an additional indicator of glycaemia that is not affected by haemoglobin level. The consistency of our primary and secondary outcomes supports the robustness of the improving trend of glycaemic control. All groups had improvement in glycaemic control over the years regardless of the cirrhosis status. The emergence of new antidiabetic agents has promoted blood glucose management in patients with T2DM. In addition, the healthcare reform from 2007 to 2014 in Hong Kong also played important roles in patient care by supporting the establishment of a comprehensive database for diabetes outcome and complication evaluation, and implementing a personalized reporting system in public primary care, which significantly reduced the incidence of complications and mortality caused by diabetes in Hong Kong [12, 15].

Interestingly, our study observed that patients with compensated cirrhosis achieved better glycaemic control than those without cirrhosis, opposite to our understanding of difficult glycaemic control in patients with cirrhosis. Difference in monitoring frequency is one of the possible reasons behind. In our study, patients with compensated cirrhosis had more frequent blood glucose measurements which indirectly reflected more intensive monitoring and management of T2DM. This finding is consistent with previous studies and guideline monitoring recomendations [16, 17, 18]. This reflects that the diagnosis of cirrhosis may strengthen subsequent patient management in clinical practice. Intensive treatment in patients with compensated cirrhosis may also correlate with the better control of glycaemic levels.

Our study also reported the changing pattern of antidiabetic medications, aligning with previous studies [14, 19]. In patients without cirrhosis and those with compensated cirrhosis, the use of metformin, GLP‐1 RAs, and SGLT‐2is increased over time, aligning with guideline recommendations and contributing to improved glycaemic control. In contrast, patients with decompensated cirrhosis remained predominantly treated with insulin, with only a modest uptake of newer agents. Sulfonylureas, once the most used medication, have been decreasingly used due to its side effects compared to the newer agents with additional health benefits [20]. Metformin remained the most commonly used first‐line treatment and is recommended to patients with compensated cirrhosis in guidelines [18], as it is associated with a reduced risk of HCC and hepatic decompensation in patients with chronic liver diseases [21, 22]. Metformin was underused in cirrhotic patients with preserved kidney function despite the rare risk of metformin‐related lactic acidosis [9, 23]. Novel medications like GLP‐1 RA and SGLT‐2i are safe and well‐tolerated in patients with compensated cirrhosis and are recommended to improve glycaemic control in this population. These drugs can also decrease the risk of various clinical outcomes. In our study, an increasing trend of prescription of these novel medications can be observed in all groups with and without cirrhosis, although the safety was not fully proven in the decompensated cirrhosis group. In a population‐based study involving individuals with T2DM and compensated cirrhosis, the incidence of decompensation events was lower in GLP‐1 RA users compared to users of DPP‐4is or sulfonylureas [24]. It can also reduce cirrhosis progression and mortality in MASLD and diabetes patients [25]. SGLT‐2i can benefit in heart failure and chronic kidney disease and may alleviate refractory ascites in patients with decompensated cirrhosis [26]. In a study in metformin users with cirrhosis, comparing with a combination treatment of DPP‐4i, treatment combined with SGLT‐2i could better enhance survival rates [27].

On the contrary, patients with decompensated cirrhosis were less likely to meet the glycaemic targets and also had the highest rate of hypoglycaemia. In this population, a distinctively different medication trajectory was observed, as insulin remained the predominant antidiabetic agent throughout the study period. This highlights the complex interplay between glycaemic control and impaired liver function in advanced liver disease [28, 29]. Cirrhosis influences glucose homeostasis by reducing insulin clearance, increasing advanced‐glycation‐end products, development of systemic hypoxia and peripheral insulin resistance, and hyperinsulinemia [30]. Furthermore, patients with cirrhosis frequently present complications such as malnutrition, sarcopenia, and renal impairment, further exacerbating glycaemic variability. The high rate of hypoglycaemia further reflects impaired gluconeogenesis, reduced hepatic glycogen storage, and altered pharmacokinetics of antidiabetic drugs in advanced liver disease [31, 32, 33]. The adoption of newer antidiabetic agents in the general T2DM population has not yet translated into similarly informed treatment paradigms for patients with decompensated cirrhosis. Insulin remains the commonest antidiabetic agent used in patients with decompensated cirrhosis, which may lead to a higher risk of hypoglycaemia. Further research is needed to refine the best approach for optimal glycaemic control in this special population.

The observed findings in glycaemic trends and medication patterns across cirrhosis stages carry several implications in clinical practice. For patients with compensated cirrhosis, better glycaemic control likely reflects more frequent monitoring and a greater awareness of diabetes management. Clinicians may consider adopting similar intensified monitoring strategies and adherence to guideline‐recommended medication algorithms. As observed in our study, the increasing use of newer agents in this population may contribute to improved outcomes, and their efficacy in reducing LRE and mortality was also reported in previous studies [34, 35]. For patients with decompensated cirrhosis, hypoglycaemia prevention should take priority over strict glycaemic targets. Besides, the roles of newer oral agents remain undefined [36]. Clinicians should exercise caution when prescribing these agents in patients with decompensated cirrhosis and prioritize insulin with careful titration [10]. To mitigate catabolism and nocturnal hypoglycaemia, supportive measures, such as a late‐night snack, may be particularly beneficial in this population [37]. The clinical decisions in compensated cirrhosis or non‐cirrhosis patients may not be appropriate for individuals with decompensated cirrhosis, given the profound alterations in drug metabolism, hypoglycaemic counter‐regulation, and nutritional status. The gap between evidence and practice in the decompensated cirrhosis group indicates the need for dedicated studies to establish the safety and efficacy of modern glucose‐lowering therapies and special targets for these patients.

The strength of the study was the sample size of over 1 million patients with T2DM for comparison of glycaemic control and medication patterns between those with and without cirrhosis over the years. However, certain limitations warrant consideration. Firstly, this study was based on data from electronic medical records, with missing data including body mass index and laboratory measurements. To address this, multiple imputation analyses were employed. Secondly, a diagnosis code‐based definition may lead to potential misclassification bias of cirrhosis, as early and mild cirrhosis may be classified as the non‐cirrhosis group. To mitigate the risk, we also used FIB‐4 and APRI to more objectively identify patients with cirrhosis, which further strengthened the identification of cirrhosis. Thirdly, the improvement in glycaemic control over the years may be partly attributable to the decreasing age at diagnosis. Hence, adjustment was made for age in regression models, yielding conclusions similar to the main results. Besides, the higher missing rate of glycaemic records in earlier calendar years may have introduced additional uncertainty into the assessment of glycaemic control and hypoglycaemia risk. It was observed that patients with decompensated cirrhosis were more likely to have missing measurements of HbA_1c_/FBG in earlier calendar periods, which may partially contribute to the observed higher rates of hypoglycaemic episodes and poorer glycaemic control. Afterwards, the missing rates were similar to those with compensated cirrhosis. Hypoglycaemia was defined by diagnosis code, which may also underestimate the event rate. However, as minor hypoglycaemia is common in patients with diabetes, the focus on clinically significant events and those resulting in hospitalization would produce more robust and meaningful results. Fourthly, this study focused on medication use; the trends in medication switching patterns still require further analysis. Lastly, unmeasured data on socio‐economic status, lifestyles, and other risk variables might impact the findings.

Glycaemic control improved over time in all T2DM patients regardless of their cirrhosis status, which was consistent with the increasing use of new antidiabetic medications. Patients with compensated cirrhosis were more likely to achieve the targets, while those with decompensated cirrhosis experienced little improvement in glycaemic control and the highest hypoglycaemia risk. With the progression of cirrhosis, metformin and new oral agents were more used in patients without cirrhosis and with compensated cirrhosis, whereas insulin was the preferred treatment for decompensated cirrhotic patients over the years. While the findings suggest comparable glycaemic control in compensated cirrhosis compared to the non‐cirrhotic group, and the unmet needs in the treatment of population with decompensated cirrhosis, research is needed to investigate the significance of glycaemic control in hepatic and extrahepatic outcomes, and to establish potentially different optimal management in populations with concomitant T2DM and cirrhosis.

## Author Contributions

**Nana Peng:** formal analysis, data curation. **Grace Lai‐Hung Wong:** conceptualization, writing – review and editing, project administration, supervision, resources, methodology. **Mary Yue Wang:** conceptualization, formal analysis, data curation, methodology, writing – original draft, writing – review and editing. **Vincent Wai‐Sun Wong:** conceptualization, supervision, project administration, writing – review and editing, funding acquisition, methodology. **Jimmy Che‐To Lai:** writing – review and editing, funding acquisition, project administration, supervision. **Terry Cheuk‐Fung Yip:** writing – review and editing, conceptualization, methodology, supervision, funding acquisition, project administration. **Sherlot Juan Song:** data curation, formal analysis.

## Funding

This work was supported by Chinese University of Hong Kong, 2025.067. Early Career Scheme by Research Grants Council, University Grants Committee, 24105223. Noncommunicable Chronic Diseases‐National Science and Technology Major Project of China, 2023ZD0508700. General Research Fund, Research Grants Council, University Grants Committee, Hong Kong, 14106824.

## Conflicts of Interest

Grace Wong has served as an advisory committee member for Gilead Sciences and Janssen, as a speaker for Abbott, Abbvie, Bristol‐Myers Squibb, Echosens, Furui, Gilead Sciences, Janssen, and Roche, and received a research grant from Gilead Sciences. Vincent Wong has served as a consultant or advisory board member for AbbVie, Boehringer Ingelheim, Echosens, Gilead Sciences, Intercept, Inventiva, Novo Nordisk, Pfizer, Sagimet Biosciences, TARGET PharmaSolutions, and Visirna; and as a speaker for Abbott, AbbVie, Echosens, Gilead Sciences, Novo Nordisk, and Unilab. He has received a research grant from Gilead Sciences and is a co‐founder of Illuminatio Medical Technology. Jimmy Lai has served as a speaker for Abbott and Gilead Sciences and on an advisory board committee for Gilead Sciences, GSK, and Boehringer Ingelheim. Terry Yip has served as a speaker and an advisory committee member for Gilead Sciences. He has received a research grant from Gilead Sciences. The other authors declare no conflicts of interest.

## Supporting information


**Figure S1:** Sensitivity analysis of haemoglobin A_1c_ and fasting blood glucose trend among patients without cirrhosis, with compensated and decompensated cirrhosis, by A. counting all records after type 2 diabetes mellitus diagnosis; B. counting only newly diagnosed, 1‐year records after diagnosis; C. counting only newly diagnosed, all records after diagnosis.
**Figure S2:** Secular trend of anti‐diabetic medications usage, counting 90 days after diagnosis in patients with type 2 diabetes, with and without cirrhosis.
**Figure S3:** Secular trend of anti‐diabetic medications usage, counting 30 days after diagnosis in patients with type 2 diabetes, with and without cirrhosis.
**Table S1:** ICD Diagnosis and Procedure Codes for definitions.
**Table S2:** Median duration and dose of each kind of anti‐diabetic medication.
**Table S3:** Frequency of blood glucose measurements of type 2 diabetes mellitus with different cirrhosis status.
**Table S4:** Time‐weighted average laboratory measurements in each period among patients without cirrhosis, with compensated cirrhosis, and with decompensated cirrhosis.
**Table S5:** Proportions of non‐cirrhosis, compensated cirrhosis, and decompensated cirrhosis patients reaching different haemoglobin A_1c_ levels.
**Table S6:** Multivariable analysis of antidiabetic medication usage and likelihood of achieving haemoglobin A_1c_ target in type 2 diabetes patients with cirrhosis, adjusted for cirrhosis status and other covariates.
**Table S7:** Multivariable analysis of antidiabetic medication usage and likelihood of achieving fasting blood glucose target in type 2 diabetes patients with cirrhosis, adjusted for cirrhosis status and other covariates.
**Table S8:** Subgroup analysis on the percentage of patients achieving the HbA_1c_ target in type 2 diabetes patients without cirrhosis, with compensated cirrhosis and decompensated cirrhosis, stratifying by age and key comorbidities.
**Table S9:** Incidence of hypoglycaemia (per 100 person‐years) among type 2 diabetes patients without cirrhosis, with compensated cirrhosis and decompensated cirrhosis over years.

## Data Availability

The data that support the findings of this study are available from the corresponding author upon reasonable request.

## References

[1] H. Sun , P. Saeedi , S. Karuranga , et al., “IDF Diabetes Atlas: Global, Regional and Country‐Level Diabetes Prevalence Estimates for 2021 and Projections for 2045,” Diabetes Research and Clinical Practice 183 (2022): 109119, 10.1016/j.diabres.2021.109119. 34879977 PMC11057359

[2] B. W. Lee , Y. H. Lee , C. Y. Park , et al., “Non‐Alcoholic Fatty Liver Disease in Patients With Type 2 Diabetes Mellitus: A Position Statement of the Fatty Liver Research Group of the Korean Diabetes Association,” Diabetes and Metabolism Journal 44, no. 3 (2020): 382–401, 10.4093/dmj.2020.0010. 32431115 PMC7332334

[3] Z. M. Younossi , P. Golabi , J. K. Price , et al., “The Global Epidemiology of Nonalcoholic Fatty Liver Disease and Nonalcoholic Steatohepatitis Among Patients With Type 2 Diabetes,” Clinical Gastroenterology and Hepatology 22, no. 10 (2024): 1999–2010.e8, 10.1016/j.cgh.2024.03.006. 38521116

[4] R. Kwok , K. C. Choi , G. L. Wong , et al., “Screening Diabetic Patients for Non‐Alcoholic Fatty Liver Disease With Controlled Attenuation Parameter and Liver Stiffness Measurements: A Prospective Cohort Study,” Gut 65, no. 8 (2016): 1359–1368, 10.1136/gutjnl-2015-309265. 25873639

[5] A. P. Kong , E. S. Lau , O. CK , et al., “Advanced Liver Fibrosis Predicts Heart Failure and Hospitalizations in People With Type 2 Diabetes: A Prospective Cohort Study From Hong Kong Diabetes Register,” Diabetes Research and Clinical Practice 202 (2023): 110825, 10.1016/j.diabres.2023.110825. 37442241

[6] D. Q. Huang , N. Noureddin , V. Ajmera , et al., “Type 2 Diabetes, Hepatic Decompensation, and Hepatocellular Carcinoma in Patients With Non‐Alcoholic Fatty Liver Disease: An Individual Participant‐Level Data Meta‐Analysis,” Lancet Gastroenterology & Hepatology 8, no. 9 (2023): 829–836, 10.1016/s2468-1253(23)00157-7. 37419133 PMC10812844

[7] K. Arvanitakis , T. Koufakis , G. Kalopitas , S. P. Papadakos , K. Kotsa , and G. Germanidis , “Management of Type 2 Diabetes in Patients With Compensated Liver Cirrhosis: Short of Evidence, Plenty of Potential,” Diabetes and Metabolic Syndrome: Clinical Research and Reviews 18, no. 1 (2024): 102935, 10.1016/j.dsx.2023.102935. 38163417

[8] P. Puri and N. Kotwal , “An Approach to the Management of Diabetes Mellitus in Cirrhosis: A Primer for the Hepatologist,” Journal of Clinical and Experimental Hepatology 12, no. 2 (2022): 560–574, 10.1016/j.jceh.2021.09.010. 35535116 PMC9077234

[9] T. C. Yip , R. N. C. Chan , V. W. Wong , et al., “Association of Metformin Use on Metabolic Acidosis in Diabetic Patients With Chronic Hepatitis B‐Related Cirrhosis and Renal Impairment,” Health Science Report 4, no. 3 (2021): e352, 10.1002/hsr2.352. PMC8358231 34401527

[10] “6. Glycemic Goals and Hypoglycemia: Standards of Care in Diabetes‐2024,” Diabetes Care 47, no. Suppl 1 (2024): S111–s125, 10.2337/dc24-S006. 38078586 PMC10725808

[11] Y. Wang , S. J. Song , Y. Jiang , et al., “Role of Noninvasive Tests in the Prognostication of Metabolic Dysfunction‐Associated Steatotic Liver Disease,” Clinical and Molecular Hepatology 31, no. Suppl (2025): S51–s75, 10.3350/cmh.2024.0246. 38934108 PMC11925434

[12] J. C. N. Chan , L. L. Lim , A. O. Y. Luk , et al., “From Hong Kong Diabetes Register to JADE Program to RAMP‐DM for Data‐Driven Actions,” Diabetes Care 42, no. 11 (2019): 2022–2031, 10.2337/dci19-0003. 31530658

[13] A. Yang , H. Wu , E. S. H. Lau , et al., “Glucose‐Lowering Drug Use, Glycemic Outcomes, and Severe Hypoglycemia: 18‐Year Trends in 0·9 Million Adults With Diabetes in Hong Kong (2002‐2019),” Lancet Regional Health–Western Pacific 26 (2022): 100509, 10.1016/j.lanwpc.2022.100509. 35789825 PMC9249907

[14] H. Yokoyama , S. I. Araki , K. Yamazaki , et al., “Trends in Glycemic Control in Patients With Insulin Therapy Compared With Non‐Insulin or no Drugs in Type 2 Diabetes in Japan: A Long‐Term View of Real‐World Treatment Between 2002 and 2018 (JDDM 66),” BMJ Open Diabetes Research & Care 10, no. 3 (2022): e002727, 10.1136/bmjdrc-2021-002727. PMC9066475 35504696

[15] J. C. N. Chan , M. Cheung , A. O. Y. Luk , et al., “A 30‐Year Case Study of Local Implementation of Global Guidelines for Data‐Driven Diabetes Management Starting With the Hong Kong Diabetes Register,” Lancet Regional Health–Western Pacific 56 (2025): 101505, 10.1016/j.lanwpc.2025.101505. 40171472 PMC11960639

[16] M. L. Volk , R. S. Tocco , J. Bazick , M. O. Rakoski , and A. S. Lok , “Hospital Readmissions Among Patients With Decompensated Cirrhosis,” American Journal of Gastroenterology 107, no. 2 (2012): 247–252, 10.1038/ajg.2011.314. 21931378 PMC3470789

[17] “EASL Clinical Practice Guidelines for the Management of Patients With Decompensated Cirrhosis,” Journal of Hepatology 69, no. 2 (2018): 406–460, 10.1016/j.jhep.2018.03.024. 29653741

[18] M. E. Rinella , B. A. Neuschwander‐Tetri , M. S. Siddiqui , et al., “AASLD Practice Guidance on the Clinical Assessment and Management of Nonalcoholic Fatty Liver Disease,” Hepatology 77, no. 5 (2023): 1797–1835, 10.1097/hep.0000000000000323. 36727674 PMC10735173

[19] Y. Z. Tan , M. H. H. Cheen , S. Y. Goh , et al., “Trends in Medication Utilization, Glycemic Control and Outcomes Among Type 2 Diabetes Patients in a Tertiary Referral Center in Singapore From 2007 to 2017,” Journal of Diabetes 11, no. 7 (2019): 573–581, 10.1111/1753-0407.12886. 30556375

[20] A. Yang , H. Wu , E. S. H. Lau , et al., “Trends in Glucose‐Lowering Drug Use, Glycemic Control, and Severe Hypoglycemia in Adults With Diabetes in Hong Kong, 2002‐2016,” Diabetes Care 43, no. 12 (2020): 2967–2974, 10.2337/dc20-0260. 33046501

[21] E. Vilar‐Gomez , R. Vuppalanchi , A. P. Desai , et al., “Long‐Term Metformin Use May Improve Clinical Outcomes in Diabetic Patients With Non‐Alcoholic Steatohepatitis and Bridging Fibrosis or Compensated Cirrhosis,” Alimentary Pharmacology & Therapeutics 50, no. 3 (2019): 317–328, 10.1111/apt.15331. 31157422

[22] X. Zhang , W. S. Harmsen , T. A. Mettler , et al., “Continuation of Metformin Use After a Diagnosis of Cirrhosis Significantly Improves Survival of Patients With Diabetes,” Hepatology 60, no. 6 (2014): 2008–2016, 10.1002/hep.27199. 24798175 PMC4218882

[23] M. J. Crowley , C. J. Diamantidis , J. R. McDuffie , et al., “Clinical Outcomes of Metformin Use in Populations With Chronic Kidney Disease, Congestive Heart Failure, or Chronic Liver Disease: A Systematic Review,” Annals of Internal Medicine 166, no. 3 (2017): 191–200, 10.7326/m16-1901. 28055049 PMC5293600

[24] T. G. Simon , E. Patorno , and S. Schneeweiss , “Glucagon‐Like Peptide‐1 Receptor Agonists and Hepatic Decompensation Events in Patients With Cirrhosis and Diabetes,” Clinical Gastroenterology and Hepatology 20, no. 6 (2022): 1382–1393.e19, 10.1016/j.cgh.2021.07.010. 34256144 PMC8743301

[25] F. Kanwal , J. R. Kramer , L. Li , et al., “GLP‐1 Receptor Agonists and Risk for Cirrhosis and Related Complications in Patients With Metabolic Dysfunction‐Associated Steatotic Liver Disease,” JAMA Internal Medicine 184, no. 11 (2024): 1314–1323, 10.1001/jamainternmed.2024.4661. 39283612 PMC11406452

[26] Y. Miyamoto , A. Honda , S. Yokose , M. Nagata , and J. Miyamoto , “The Effects of SGLT2 Inhibitors on Liver Cirrhosis Patients With Refractory Ascites: A Literature Review,” Journal of Clinical Medicine 12, no. 6 (2023): 2253, 10.3390/jcm12062253. 36983252 PMC10056954

[27] S. Saffo , D. E. Kaplan , N. Mahmud , et al., “Impact of SGLT2 Inhibitors in Comparison With DPP4 Inhibitors on Ascites and Death in Veterans With Cirrhosis on Metformin,” Diabetes, Obesity & Metabolism 23, no. 10 (2021): 2402–2408, 10.1111/dom.14488. PMC8429193 34227216

[28] A. Mantovani , A. Taverna , D. Cappelli , et al., “Long‐Term Adverse Effect of Liver Stiffness on Glycaemic Control in Type 2 Diabetic Patients With Nonalcoholic Fatty Liver Disease: A Pilot Study,” International Journal of Molecular Sciences 23, no. 20 (2022): 12481, 10.3390/ijms232012481. 36293337 PMC9604384

[29] F. S. Yen , M. C. Hou , J. S. Liu , C. C. Hsu , and C. M. Hwu , “Severe Hypoglycemia in Patients With Liver Cirrhosis and Type 2 Diabetes,” Front Med (Lausanne) 9 (2022): 962337, 10.3389/fmed.2022.962337. 36687427 PMC9845885

[30] L. Elkrief , P. E. Rautou , S. Sarin , D. Valla , V. Paradis , and R. Moreau , “Diabetes Mellitus in Patients With Cirrhosis: Clinical Implications and Management,” Liver International 36, no. 7 (2016): 936–948, 10.1111/liv.13115. 26972930

[31] F. Meyer , K. Bannert , M. Wiese , et al., “Molecular Mechanism Contributing to Malnutrition and Sarcopenia in Patients With Liver Cirrhosis,” International Journal of Molecular Sciences 21, no. 15 (2020): 5357, 10.3390/ijms21155357. 32731496 PMC7432938

[32] A. J. Scheen , “Pharmacokinetics in Patients With Chronic Liver Disease and Hepatic Safety of Incretin‐Based Therapies for the Management of Type 2 Diabetes Mellitus,” Clinical Pharmacokinetics 53, no. 9 (2014): 773–785, 10.1007/s40262-014-0157-y. 25091053

[33] K. K. Changani , R. Jalan , I. J. Cox , et al., “Evidence for Altered Hepatic Gluconeogenesis in Patients With Cirrhosis Using In Vivo 31‐Phosphorus Magnetic Resonance Spectroscopy,” Gut 49, no. 4 (2001): 557–564, 10.1136/gut.49.4.557. 11559655 PMC1728472

[34] F. S. Yen , M. C. Hou , J. Cheng‐Chung Wei , et al., “Glucagon‐Like Peptide‐1 Receptor Agonist Use in Patients With Liver Cirrhosis and Type 2 Diabetes,” Clinical Gastroenterology and Hepatology 22, no. 6 (2024): 1255–1264.e18, 10.1016/j.cgh.2023.06.004. 37331413

[35] X. Mao , X. Zhang , R. Lai , et al., “Glucagon‐Like Peptide 1 Receptor Agonist and Reduced Liver and Non‐Liver Complications in Adults With Type 2 Diabetes and Metabolic Dysfunction‐Associated Steatotic Liver Disease: A Target Trial Emulation Study,” Clinical and Molecular Hepatology 31, no. 3 (2025): 1084–1099, 10.3350/cmh.2024.1096. 40268291 PMC12260620

[36] R. Loomba , M. F. Abdelmalek , M. J. Armstrong , et al., “Semaglutide 2·4 Mg Once Weekly in Patients With Non‐Alcoholic Steatohepatitis‐Related Cirrhosis: A Randomised, Placebo‐Controlled Phase 2 Trial,” Lancet Gastroenterology & Hepatology 8, no. 6 (2023): 511–522, 10.1016/s2468-1253(23)00068-7. 36934740 PMC10792518

[37] Z. Han , R. Li , Z. Zhong , Y. Piao , and R. Guo , “Clinical Effect of Nighttime Snacking on Patients With Hepatitis B Cirrhosis,” Frontiers in Nutrition 9 (2022): 999462, 10.3389/fnut.2022.999462. 36704800 PMC9871573

