# Supplementary material for: Secular Trend in Glycaemic Management in Type 2 Diabetes Patients With and Without Cirrhosis Between 2000 and 2023: A Territory‐Wide Cohort Study
Source: Aliment Pharmacol Ther. 2026 May 5;64(3):356–66. doi: 10.1111/apt.70705 (PMC13356366; doi:10.1111/apt.70705)
Supplement: Supplementary file 1 — Figure S1: Sensitivity analysis of haemoglobin A1c and fasting blood glucose trend among patients without cirrhosis, with compensated and decompensated cirrhosis, by A. counting all records after type 2 diabetes mellitus diagnosis; B. counting only newly diagnosed, 1‐year records after diagnosis; C. counting only newly diagnosed, all records after diagnosis. Figure S2: Secular trend of anti‐diabetic medications usage, counting 90 days after diagnosis in patients with type 2 diabetes, with and without cirrhosis. Figure S3: Secular trend of anti‐diabetic medications usage, counting 30 days after diagnosis in patients with type 2 diabetes, with and without cirrhosis. Table S1: ICD Diagnosis and Procedure Codes for definitions. Table S2: Median duration and dose of each kind of anti‐diabetic medication. Table S3: Frequency of blood glucose measurements of type 2 diabetes mellitus with different cirrhosis status. Table S4: Time‐weighted average laboratory measurements in each period among patients without cirrhosis, with compensated cirrhosis, and with decompensated cirrhosis. Table S5: Proportions of non‐cirrhosis, compensated cirrhosis, and decompensated cirrhosis patients reaching different haemoglobin A1c levels. Table S6: Multivariable analysis of antidiabetic medication usage and likelihood of achieving haemoglobin A1c target in type 2 diabetes patients with cirrhosis, adjusted for cirrhosis status and other covariates. Table S7: Multivariable analysis of antidiabetic medication usage and likelihood of achieving fasting blood glucose target in type 2 diabetes patients with cirrhosis, adjusted for cirrhosis status and other covariates. Table S8: Subgroup analysis on the percentage of patients achieving the HbA1c target in type 2 diabetes patients without cirrhosis, with compensated cirrhosis and decompensated cirrhosis, stratifying by age and key comorbidities. Table S9: Incidence of hypoglycaemia (per 100 person‐years) among type 2 diabetes patients without cirrhos [file APT-64-356-s001.docx]

**Secular trend in glycaemic management in type 2 diabetes patients with and without cirrhosis between 2000 and 2023: A territory-wide cohort study**

Mary Yue Wang, Sherlot Juan Song, Nana Peng, Grace Lai-Hung Wong, Vincent Wai-Sun Wong, Jimmy Che-To Lai, Terry Cheuk-Fung Yip

**TABLE OF CONTENTS**

eMethods...………………………………………..……………….………………….2

Figure S1……...……………………………………………….……...……………....4

Figure S2…………...…………………...….………………………………………....5

Figure S3…………………………………....…………………………………...…....6

Table S1……………………………….…….………………………………………...7

Table S2……………………………………..………………………………………...9

Table S3………………………..…………….…………………………………….…10

Table S4……..……………………………….……………………………………….11

Table S5…………..………………………….……………………………………….13

Table S6……..……………………………….……………………………………….14

Table S7……..……………………………….……………………………………….15

Table S8……..……………………………….……………………………………….16

Table S9……..……………………………….……………………………………….17

Reference……….………………………………………………………………….…18

**eMethods**

**Introduction of Clinical Data Analysis and Reporting System (CDARS)**

CDARS is an electronic healthcare database created in 1993 and managed by the Hospital Authority, Hong Kong.^1^ CDARS covers data on approximately 80% of the local population (about 7.4 million residents), containing patients from 43 public hospitals and outpatients from 122 public general and specialist clinics in Hong Kong. The ethnicity is mainly Han Chinese, accounting for 92% of the included population.^2, 3^ This database can provide data including demographics, death status, diagnoses, procedures, laboratory measurements, and medication prescription records from public hospitals and clinics.

**Definitions of comorbidities and aetiology**

The body mass index (BMI), calculated as weight in kilograms divided by the square of the height in meters, was used to classify patients into three subgroups: normal (BMI <23 kg/m^2^), overweight (BMI ≥23 to <25 kg/m^2^), and obese (BMI ≥25 kg/m^2^). ^4^

Hypertension was defined as the usage of any anti-hypertensive medications and International Classification of Diseases (ICD) diagnosis codes of hypertension listed in **Table S1**.

Chronic kidney disease (CKD) was categorized by estimated glomerular filtration rate (eGFR), categorized as: stage 1 [≥90 mL/min/1.73 m^2^], stage 2 [60–89 mL/min/1.73 m^2^], stage 3 [30–59 mL/min/1.73 m^2^], stage 4 [16–29 mL/min/1.73 m^2^], stage 5 [<15 mL/min/1.73 m^2^].

Dyslipidaemia was defined by the use of lipid-lowering agents, triglyceride ≥1.7 mmol/L, high-density lipoprotein-cholesterol (HDL-C) <1.03 mmol/L in males or <1.29 mmol/L in females, low-density lipoprotein-cholesterol (LDL-C) ≥4.1 mmol/L, and/or the ICD diagnosis codes for hyperlipidaemia listed in **Table S1**.

Hepatitis virus infection, including infection of hepatitis B virus (HBV), hepatitis C virus (HCV) and hepatitis D virus (HDV), was defined by the ICD diagnosis codes listed in **Table S1**, and the use of antiviral medication.

Excessive alcohol usage was defined by the ICD diagnosis codes listed in **Table S1**.

Hypoglycaemia was defined by the ICD diagnosis codes listed in **Table S1**, and then manually excluded diagnosis not related with type 2 diabetes mellitus (T2DM).

Major adverse cardiovascular event (MACE) was defined as 4-point MACE (myocardial infarction, stroke, heart failure, and cardiovascular-related death), identified by ICD diagnosis codes listed in **Table S1**, and patients who received percutaneous coronary intervention or coronary artery bypass grafting with typical 99th percentile upper reference limit of troponin I (>40 ng/L) or troponin T (>14 ng/L) levels were also defined as MACE.

**Calculation of fibrosis-4 (FIB-4) and AST to platelet ratio index (APRI) indexes**

The FIB-4 index was calculated as (age [years] × aspartate aminotransferase [AST] [IU/L]) / (platelet counts [×10^9^/L]) × alanine aminotransferase [ALT] ^1/2^ [IU/L]). APRI was calculated as (AST [IU/L]/upper limit of normal)/platelet counts (×10^9^/L). Decompensated cirrhosis was defined by the presence of hepatic decompensation.

**Imputed variables (percentage of missing data)**

Only patients with at least one record of HbA_1c_ and FBG were included for imputation. The imputed variables (percentage of missing data) were BMI (43.0%), total bilirubin (8.5%), total cholesterol (4.3%), platelets (18.8%), albumin (8.3%), ALT (5.4%), AST (73.1%), and creatinine (3.8%s).

**Figure S1.** Sensitivity analysis of haemoglobin A_1c_ and fasting blood glucose trend among patients without cirrhosis, with compensated and decompensated cirrhosis, by A. counting all records after type 2 diabetes mellitus diagnosis; B. counting only newly diagnosed, 1-year records after diagnosis; C. counting only newly diagnosed, all records after diagnosis.
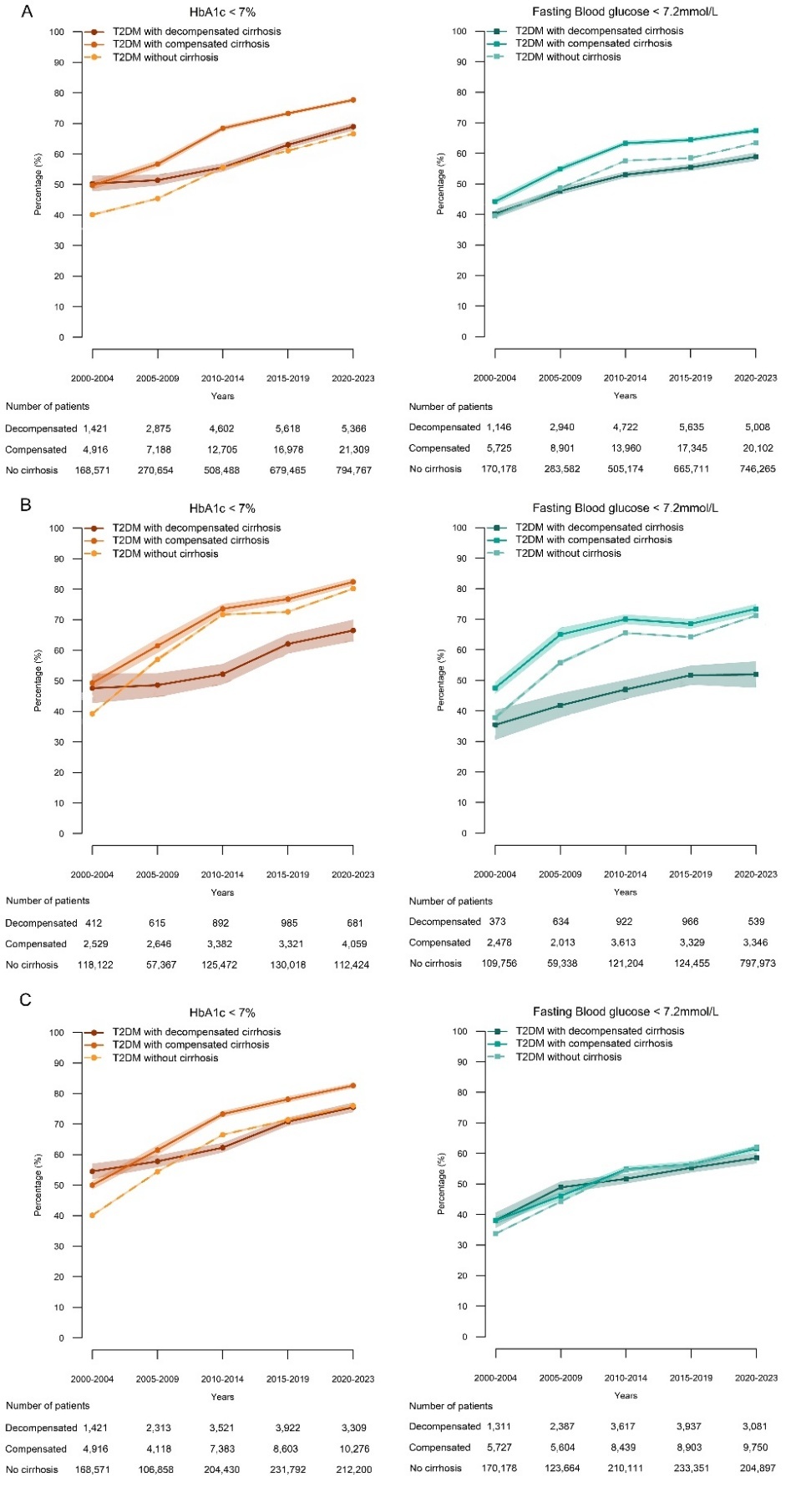


Abbreviation: HbA_1c_, haemoglobin A_1c_.

**Figure S2.** Secular trend of anti-diabetic medications usage, counting 90 days after diagnosis in patients with type 2 diabetes, with and without cirrhosis.


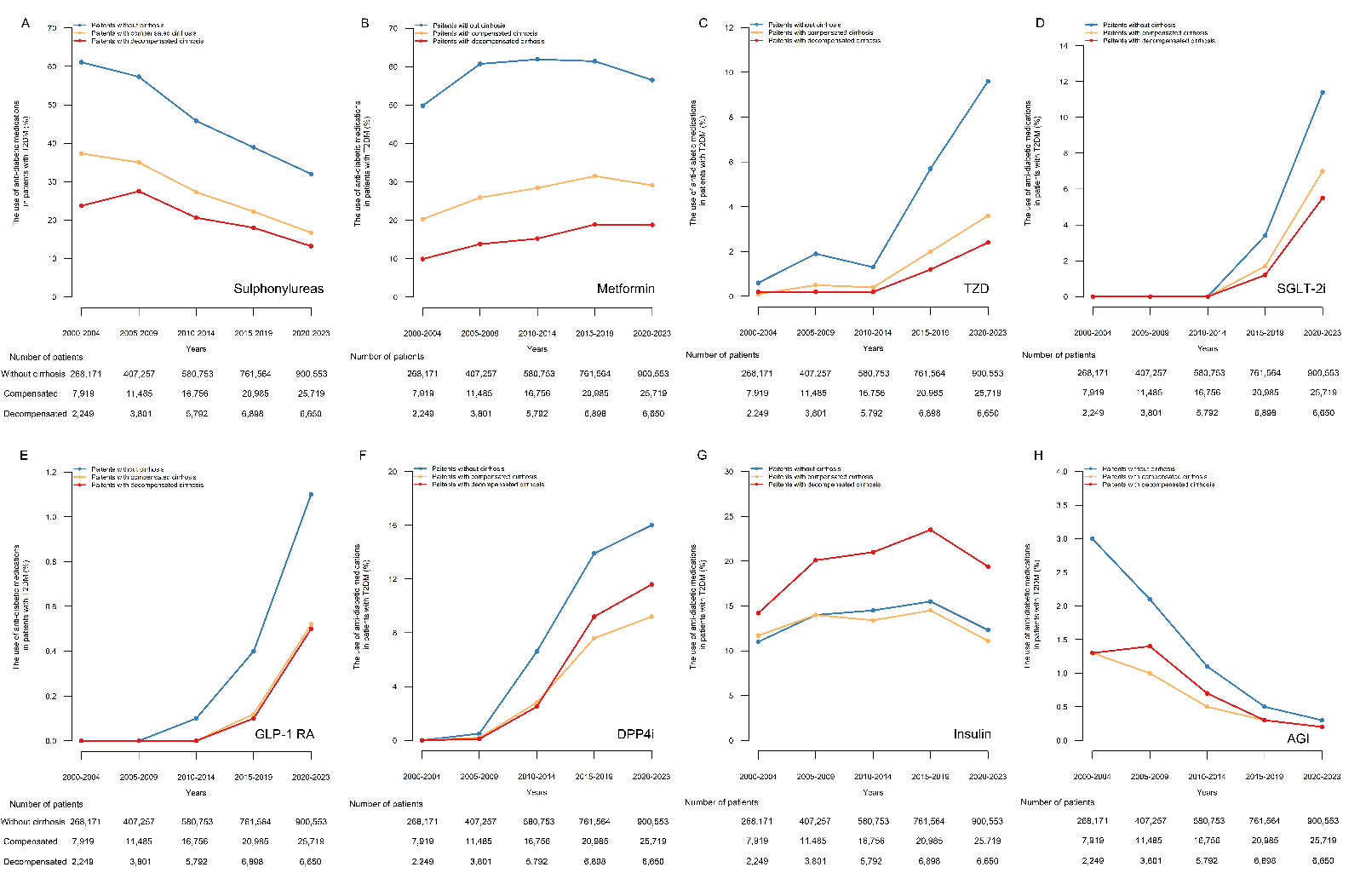
Abbreviation: TZD, thiazolidinedione; SGLT-2i, sodium-glucose cotransporter 2 inhibitor; GLP-1A, glucose like peptide-1 receptor agonists; DPP-4i, dipeptidyl peptidase-4 inhibitor; AGI, Acarbose.

**Figure S3.** Secular trend of anti-diabetic medications usage, counting 30 days after diagnosis in patients with type 2 diabetes, with and without cirrhosis.


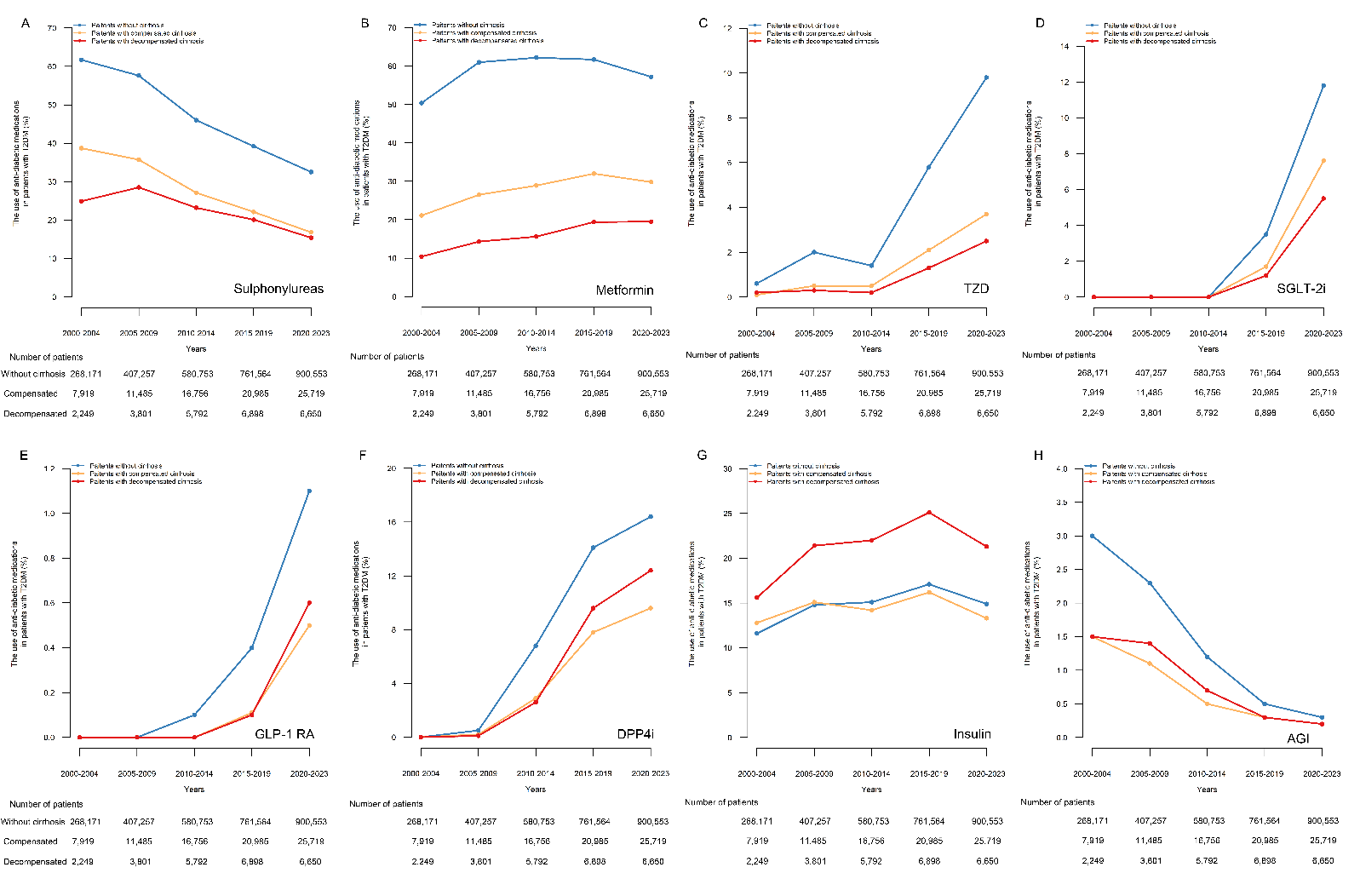


Abbreviation: TZD, thiazolidinedione; SGLT-2i, sodium-glucose cotransporter 2 inhibitor; GLP-1A, glucose like peptide-1 receptor agonists; DPP-4i, dipeptidyl peptidase-4 inhibitor.

**Table S1**. ICD Diagnosis and Procedure Codes for definitions.

| Disease or Procedure | ICD codes |
| --- | --- |
| Type 1 diabetes | 250.01, 250.03, 250.11, 250.13, 250.21, 250.23 250.31, 250.33, 250.41, 250.43, 250.51, 250.53, 250.61, 250.63, 250.71, 250.73, 250.81, 250.83, 250.91, 250.93 |
| Type 2 diabetes | 250.00, 250.02, 250.10, 250.12, 250.20, 250.22, 250.30, 250.32, 250.40, 250.42, 250.50, 250.52, 250.60, 250.62, 250.70, 250.72, 250.80, 250.82, 250.90, 250.92 |
| Liver cirrhosis | 571.2, 571.5, 571.51, 571.52, 571.53, 571.6, 571 |
| Hepatic complications |  |
| Ascites | 789.5 |
| Spontaneous bacterial peritonitis | 567.2:9 |
| Oesophageal variceal bleeding | 456.0; 456.20; 571.5:2; 573.9:2, 42.33:3, 42.33:6, 42.33:13 |
| Oesophageal variceal without bleeding | 456.1, 456.21, 571.5:1, 42.33:1, 42.33:5, 42.33:10, 42.91:0 |
| Gastric variceal bleeding | 456.8:1; 456.8:2, 43.41:1 |
| Gastric variceal without bleeding | 456.21, 456.1, 43.41:3 |
| Hepatic encephalopathy | 572.2 |
| Hepatorenal syndrome | 572.4 |
| Hepatopulmonary syndrome | 573.8:13 |
| Portal hypertension | 572.3, 537.89:8 |
| Comorbidities |  |
| Hypertension | 401, 401.0, 401.1, 401.9 |
| Dyslipidaemia | 272.0-272.4 |
| Aetiology of liver diseases |  |
| Excessive use of alcohol | 291, 291.0, 291.1, 291.2, 291.3, 291.4 291.5, 291.8, 291.81, 291.89, 291.9; 303, 303.0, 303.00, 303.01, 303.02, 303.03 303.90, 303.91, 303.92, 303.93; 305, 305.00, 305.01, 305.02, 305.03; 357.5; 425.5; 535.30, 535.31; 571.0, 571.1 571.2, 571.3; 655.4; 760.71; V11.3; E860.0 |
| Hepatitis B | 070.20-070.23; 070.30-070.33; V02.61 |
| Hepatitis C | 070.41, 070.44; 070.51, 070.54; V02.62 |
| Hepatitis D | 070.42,070.52 |
| Major adverse cardiovascular events |  |
| Myocardial infraction | 410; Valve replacement/Coronary artery bypass grafting: 36.01-07, 36.10-16, 36.99 |
| Heart failure | 428 |
| Stroke | 430, 431, 432, 433, 434, 436 |
| Cardiovascular death | I00-13, I33, I40, I50, I26-28, I34-38, I42-49, I51, I60-78, R98-99, R960-961, G45 |

Abbreviation: ICD, International Classification of Diseases.

**Table S2.** Median duration and dose of each kind of anti-diabetic medication.

| Medication | Median duration (years, IQR) | Median Defined Daily Dosage (mg, IQR) |
| --- | --- | --- |
| Sulfonylureas | 6.25 (1.99, 12.30) |  |
| Acetohexamide | 1.33 (0.01, 1.49) | 1000 (625, 1000) |
| Chlorpropamide | 0.64 (0.06, 2.20) | 250 (250, 500) |
| Glibenclamide | 3.57 (0.79, 7.07) | 10 (5, 10) |
| Gliclazide | 4.74 (1.16, 10.10) | 120 (80, 160) |
| Glimepiride | 3.25 (0.99, 6.68) | 4 (2, 4) |
| Glipizide | 1.24 (0.24, 4.05) | 10 (5, 10) |
| Tolbutamide | 0.74 (0.12, 2.45) | 1500 (1000, 1500) |
| Metformin | 6.63 (2.52, 12.30) |  |
| Actosmet | 1.75 (0.61, 3.16) | 30 (30, 30) |
| Diabetmin | 0.51 (0.51, 0.51) | 850 (850, 850) |
| Glucophage | 0.27 (0.27, 0.27) | 2 (2, 2) |
| Guamet | 0.27 (0.27, 0.27) | 500 (500-500) |
| Kombiglyze | 0.54 (0.03, 2.00) | 5 (5-5) |
| Metformin | 5.15 (1.98, 11.00) | 1000 (750-1500) |
| TZD | 2.42 (0.84, 4.56) |  |
| Actosmet | 1.75 (0.61, 3.16) | 30 (30, 30) |
| Oseni | 1.78 (0.58, 4.28) | 25 (25, 25) |
| Pioglitazone | 2.42 (0.86, 4.50) | 15 (15, 30) |
| Rosiglitazone | 0.92 (0.31, 2.17) | 4 (4, 4) |
| α-glucosidase inhibitors | 1.27 (0.31, 3.53) | 150 (150, 300) |
| SGLT-2i | 1.73 (0.93, 3.33) |  |
| Canagliflozin | 0.89 (0.36, 1.35) | 100 (100, 100) |
| Dapagliflozin | 1.32 (0.58, 2.61) | 10 (10, 10) |
| Empagliflozin | 1.87 (0.73, 3.47) | 10 (10, 25) |
| Ertugliflozin | 0.35 (0.25, 0.63) | 15 (15, 15) |
| GLP-1 RA | 2.03 (0.81, 3.55) |  |
| Dulaglutide | 1.53 (0.61, 2.88) | 2.25 (0.56, 2.25) |
| Exenatide | 0.83 (0.38, 1.34) | 4 (4, 4) |
| Liraglutide | 1.25 (0.54, 2.48) | 18 (18, 18) |
| Lixisenatide | 0.44 (0.23, 1.11) | 400 (400, 400) |
| Semaglutide | 1.09 (0.46, 2.13) | 0.25 (0.06, 1.00) |
| DPP-4i | 3.15 (1.09, 6.07) |  |
| Alogliptin | 2.16 (0.81, 3.82) | 25 (25, 25) |
| Kombiglyze | 0.54 (0.03, 2.00) | 5 (5, 5) |
| Linagliptin | 1.89 (0.60, 3.97) | 5 (5, 5) |
| Oseni | 1.78 (0.58, 4.28) | 25 (25, 25) |
| Saxagliptin | 1.38 (0.46, 2.92) | 5 (5, 5) |
| Sitagliptin | 2.40 (0.59, 5.26) | 100 (100, 100) |
| Trazenta | 0.27 (0.27, 0.27) | 5 (5, 5) |
| Vildagliptin | 2.04 (0.63, 4.35) | 50 (50, 100) |

**Table S3.** Frequency of blood glucose measurements of type 2 diabetes mellitus with different cirrhosis status.

|  | Median number of HbA_1c_ measurements per year | Median number of fasting blood glucose measurements per year |
| --- | --- | --- |
| Patients without cirrhosis  (N = 1,143,033) | 1.38 (0.81, 1.97) | 1.10 (0.70, 1.66) |
| Patients with compensated cirrhosis  (N = 45,208) | 1.46 (0.80, 2.30) | 1.36 (0.76, 2.38) |
| Patients with decompensated cirrhosis  (N = 17,992) | 1.43 (0.84, 2.14) | 1.30 (0.76, 2.14) |

Abbreviation: HbA_1c_, haemoglobin A_1c_.

**Table S4**. Time-weighted average laboratory measurements in each period among patients without cirrhosis, with compensated cirrhosis, and with decompensated cirrhosis.

| Characteristics | Patients without cirrhosis^ | | | | | Patients with compensated cirrhosis^#^ | | | | | Patients with decompensated cirrhosis^@^ | | | | | *p* value^$^ | *p* value^%^ |
| --- | --- | --- | --- | --- | --- | --- | --- | --- | --- | --- | --- | --- | --- | --- | --- | --- | --- |
|  | **2000-04**  **N=274,527** | **2005-09**  **N=414,676** | **2010-14**  **N=587,307** | **2015-19**  **N=765,409** | **2020-23**  **N=900,553** | **2000-04**  **N=7,917** | **2005-09**  **N=11,485** | **2010-14**  **N=16,756** | **2015-19**  **N=20,985** | **2020-23**  **N=25,719** | **2000-04**  **N=2,249** | **2005-09**  **N=3,800** | **2010-14**  **N=5,792** | **2015-19**  **N=6,898** | **2020-23**  **N=6,650** |  |  |
| Albumin (g/L) | 39.2 ± 5.9 | 39.1 ± 6.4 | 40.0 ± 5.9 | 39.6 ± 5.8 | 38.8 ± 5.9 | 33.7 ± 7.5 | 34.5 ± 7.9 | 35.3 ± 7.7 | 35.4 ± 7.8 | 34.9 ± 8.0 | 29.0 ± 7.7 | 30.3 ± 7.9 | 31.3 ± 8.0 | 33.3 ± 7.9 | 31.3 ± 7.9 | <0.001 | <0.001 |
| Missing (%) | 27.0 | 23.8 | 13.0 | 13.2 | 16.2 | 3.8 | 4.3 | 4.3 | 5.6 | 6.9 | 13.2 | 1.9 | 2.2 | 3.3 | 6.1 |  |  |
| ALT (U/L) | 20 (14,30) | 22 (16,31) | 21 (15,30) | 22 (16,30) | 22 (17,31) | 26 (17,46) | 25 (17,42) | 24 (16,38) | 24 (17,39) | 26 (18,39) | 38 (24,65) | 34 (22,57) | 31 (20,51) | 28 (19,46) | 27 (18,43) | 0.282 | <0.001 |
| Missing (%) | 27.7 | 23.8 | 11.6 | 9.6 | 12.1 | 4.1 | 4.4 | 4.3 | 5.3 | 6.7 | 13.3 | 1.9 | 2.2 | 3.3 | 6.1 |  |  |
| AST (U/L) | 22 (17,28) | 23 (18,30) | 23 (18,30) | 23 (18,31) | 25 (20,33) | 39 (26,70) | 36 (25,64) | 34 (25,60) | 35 (24,65) | 38 (26,71) | 50 (32,84) | 48 (31,82) | 44 (28,78) | 40 (26,66) | 41 (27,71) | 0.817 | 0.006 |
| Missing (%) | 74.7 | 75.6 | 76.4 | 79.2 | 76.7 | 14.9 | 28.6 | 28.5 | 38.1 | 33.8 | 48.7 | 44.6 | 42.0 | 47.6 | 41.9 |  |  |
| Creatinine (μmol/L) | 88 (74,108) | 84 (69,105) | 80  (66,99) | 79  (65,98) | 78  (64,97) | 101 (81,143) | 94 (76,130) | 91 (73,123) | 89 (72,121) | 87 (69,116) | 115 (86,182) | 115 (85,192) | 112 (82,192) | 112 (80,193) | 110 (79,190) | 0.793 | <0.001 |
| Missing (%) | 24.4 | 20.3 | 9.2 | 9.3 | 11.0 | 7.1 | 6.9 | 6.9 | 8.2 | 9.5 | 16.4 | 6.0 | 5.6 | 7.0 | 9.5 |  |  |
| HDL-C (mmol/L) | 1.3 ± 0.4 | 1.2 ± 0.3 | 1.3 ± 0.3 | 1.3 ± 0.3 | 1.3 ± 0.3 | 1.3 ± 0.4 | 1.2 ± 0.4 | 1.3 ± 0.4 | 1.3 ± 0.4 | 1.3 ± 0.4 | 1.3 ± 0.5 | 1.2 ± 0.4 | 1.2 ± 0.4 | 1.2 ± 0.4 | 1.2 ± 0.4 | <0.001 | <0.001 |
| Missing (%) | 52.5 | 36.2 | 12.7 | 11.4 | 12.4 | 56.7 | 33.8 | 19.6 | 18.4 | 18.6 | 66.5 | 37.2 | 22.6 | 19.9 | 22.0 |  |  |
| LDL-C (mmol/L) | 3.1 ± 0.9 | 2.9 ± 0.8 | 2.5 ± 0.8 | 2.3 ± 0.7 | 2.2 ± 0.7 | 2.8 ± 1.0 | 2.6 ± 0.9 | 2.4 ± 0.8 | 2.2 ± 0.8 | 2.1 ± 0.8 | 2.6 ± 1.0 | 2.4 ± 1.0 | 2.4 ± 1.0 | 2.2 ± 0.8 | 2.1 ± 0.8 | 0.585 | <0.001 |
| Missing (%) | 53.2 | 36.6 | 12.9 | 11.5 | 12.6 | 57.1 | 34.0 | 19.7 | 18.5 | 18.9 | 66.9 | 37.5 | 22.7 | 20.0 | 22.2 |  |  |
| Platelets (x10^9^/L) | 253 ± 81 | 256 ± 81 | 238 ± 73 | 241 ± 74 | 248 ± 76 | 192 ± 89 | 197 ± 90 | 185 ± 82 | 190 ± 85 | 195 ± 88 | 144 ± 93 | 157 ± 99 | 158 ± 95 | 166 ± 98 | 172 ± 99 | 0.073 | 0.341 |
| Missing (%) | 33.6 | 26.9 | 24.8 | 23.5 | 26.6 | 4.2 | 5.0 | 5.3 | 6.9 | 8.1 | 13.3 | 1.9 | 2.6 | 3.7 | 6.5 |  |  |
| Total bilirubin (μmol/L) | 10 (7,13) | 11 (8,14) | 11 (8,14) | 10 (8,13) | 10 (7,13) | 13 (9,19) | 13 (9,19) | 13 (9,18) | 12 (9,17) | 11 (8,16) | 26 (14,55) | 25 (14,54) | 21 (12,47) | 19 (11,41) | 17 (10,35) | 0.460 | <0.001 |
| Missing (%) | 27.9 | 23.9 | 13.1 | 13.4 | 16.4 | 3.9 | 4.3 | 4.3 | 5.7 | 6.9 | 13.2 | 1.8 | 2.2 | 3.4 | 6.1 |  |  |
| Total cholesterol (μmol/L) | 5.1 ± 1.0 | 4.9 ± 1.0 | 4.4 ± 1.0 | 4.2 ± 0.9 | 4.2 ± 0.9 | 4.7 ± 1.2 | 4.5 ± 1.1 | 4.2 ± 1.1 | 4.1 ± 1.0 | 4.0 ± 1.0 | 4.3 ± 1.2 | 4.2 ± 1.2 | 4.1 ± 1.2 | 3.9 ± 1.1 | 3.9 ± 1.1 | <0.001 | <0.001 |
| Missing (%) | 41.7 | 34.4 | 12.5 | 11.3 | 14.2 | 36.0 | 30.6 | 19.3 | 18.1 | 22.2 | 52.2 | 32.8 | 22.1 | 19.7 | 24.3 |  |  |
| Triglyceride(μmol/L) | 1.9 ± 1.3 | 1.7 ± 1.2 | 1.5 ± 1.0 | 1.6 ± 0.9 | 1.5 ± 0.9 | 1.6 ± 1.2 | 1.5 ± 1.1 | 1.4 ± 1.1 | 1.4 ± 0.9 | 1.4 ± 0.9 | 1.4 ± 1.0 | 1.4 ± 1.0 | 1.3 ± 0.8 | 1.3 ± 0.8 | 1.3 ± 0.8 | <0.001 | 0.532 |
| Missing (%) | 42.2 | 34.5 | 12.6 | 11.3 | 12.9 | 37.1 | 30.9 | 19.4 | 18.2 | 19.4 | 53.5 | 33.6 | 22.1 | 19.7 | 22.5 |  |  |

Abbreviation: ALT, alanine aminotransferase. AST, aspartate aminotransferase. HDL-C, high-density lipoprotein cholesterol. LDL-C, low-density lipoprotein cholesterol.

Continuous variables were presented by mean ± standard deviation or median (25^th^ percentile, 75^th^ percentile), as appropriate.

^ All p values <0.001 for linear trend in consecutive periods in patients without cirrhosis.

^#^ P value of AST = 0.226, p value of HDL-C = 0.013, and other p values <0.001 for linear trend in consecutive periods in patients with compensated cirrhosis.

^@^ All p values <0.001 for linear trend in consecutive periods in patients with decompensated cirrhosis.

^$^P value compared the overall difference in mean and proportion across patients without cirrhosis, with compensated cirrhosis, and with decompensated cirrhosis.

^%^P value compared the difference in linear trend across patients without cirrhosis, with compensated cirrhosis, and with decompensated cirrhosis.

**Table S5**. Proportions of non-cirrhosis, compensated cirrhosis, and decompensated cirrhosis patients reaching different haemoglobin A_1c_ levels.

| HbA_1c_ levels | Patients without cirrhosis | | | | | Patients with compensated cirrhosis | | | | | Patients with decompensated cirrhosis | | | | |
| --- | --- | --- | --- | --- | --- | --- | --- | --- | --- | --- | --- | --- | --- | --- | --- |
|  | 2000-04 | 2005-09 | 2010-14 | 2015-19 | 2020-23 | 2000-04 | 2005-09 | 2010-14 | 2015-19 | 2020-23 | 2000-04 | 2005-09 | 2010-14 | 2015-19 | 2020-23 |
| HbA_1c_ <6.5% | 27,158 (23.0%) | 56,706 (25.8%) | 125,913 (29.4%) | 182,508 (31.6%) | 250,442 (36.1%) | 847 (33.5%) | 1,745 (37.6%) | 3,889 (45.0%) | 5,921 (51.0%) | 8,338 (55.7%) | 137 (33.3%) | 374 (32.8%) | 689 (35.6%) | 1,117 (42.4%) | 1,284 (47.6%) |
| HbA_1c_ <7% | 46,327 (39.2%) | 96,655 (44.0%) | 235,383 (54.9%) | 342,822 (59.4%) | 457,034 (65.9%) | 1,247 (49.3%) | 2,524 (54.4%) | 5,729 (66.3%) | 8,260 (71.1%) | 11,353 (75.8%) | 196 (47.6%) | 544 (47.8%) | 1,008 (52.1%) | 1,541 (58.5%) | 1,745 (64.6%) |
| HbA_1c_ <8% | 81,951 (69.4%) | 163,910 (74.6%) | 362,000 (84.4%) | 501,905 (87.0%) | 626,969 (90.4%) | 1,851 (73.2%) | 3,698 (79.7%) | 7,540 (87.3%) | 10,525 (90.6%) | 13,870 (92.6%) | 273 (66.3%) | 769 (67.5%) | 1,466 (75.7%) | 2,150 (81.6%) | 2,325 (86.1%) |

Abbreviation: HbA_1c_, haemoglobin A_1c_.

**Table S6.** Multivariable analysis of antidiabetic medication usage and likelihood of achieving haemoglobin A_1c_ target in type 2 diabetes patients with cirrhosis, adjusted for cirrhosis status and other covariates.

| Medication | OR (95% CI) of users | *P* value |
| --- | --- | --- |
| Sulfonylureas | 0.29 (0.28-0.29) | <0.001 |
| Metformin | 0.29 (0.28-0.29) | <0.001 |
| TZD | 0.41 (0.41-0.42) | <0.001 |
| α-glucosidase inhibitors | 0.44 (0.42-0.46) | <0.001 |
| SGLT-2i | 0.45 (0.44-0.46) | <0.001 |
| GLP-1 RA | 0.58 (0.55-0.61) | <0.001 |
| DPP-4i | 0.35 (0.35-0.36) | <0.001 |
| Insulin | 0.33 (0.33-0.33) | <0.001 |
| ACEI | 1.03 (1.02-1.04) | <0.001 |
| ARB | 1.10 (1.09-1.11) | <0.001 |
| Beta blockers | 1.16 (1.15-1.17) | <0.001 |
| Calcium channel blockers | 1.19 (1.18-1.20) | <0.001 |
| Thiazide diuretics | 1.05 (1.03-1.06) | <0.001 |
| Statin | 1.07 (1.06-1.08) | <0.001 |
| Other lipid-lowering agents | 1.08 (1.06-1.09) | <0.001 |
| Aspirin | 1.05 (1.04-1.05) | <0.001 |

Abbreviation: TZD, thiazolidinedione. SGLT-2i, sodium-glucose co-transporter-2 inhibitors. GLP-1 RA, glucagon-like peptide-1 agonist. DPP-4i, dipeptidyl peptidase-4 inhibitors. ACEI, angiotensin converting enzyme inhibitors. ARB, angiotensin receptor blockers.

All medication was analysed independently, compared to non-users. Adjustment for cirrhosis status (compensated cirrhosis, decompensated cirrhosis), age, gender, body mass index, type 2 diabetes mellitus duration, hypertension, dyslipidaemia, hepatitis virus infection, excessive alcohol intake, estimated glomerular filtration rate (eGFR), total bilirubin, total cholesterol, platelet, albumin, alanine aminotransferase and aspartate aminotransferase.

**Table S7.** Multivariable analysis of antidiabetic medication usage and likelihood of achieving fasting blood glucose target in type 2 diabetes patients with cirrhosis, adjusted for cirrhosis status and other covariates.

| Medication | OR (95% CI) of users | *P* value |
| --- | --- | --- |
| Sulfonylureas | 0.44 (0.43-0.44) | <0.001 |
| Metformin | 0.41 (0.41-0.42) | <0.001 |
| TZD | 0.74 (0.73-0.75) | <0.001 |
| α-glucosidase inhibitors | 0.62 (0.60-0.64) | <0.001 |
| SGLT-2i | 0.89 (0.87-0.90) | <0.001 |
| GLP-1 RA | 0.79 (0.75-0.82) | <0.001 |
| DPP-4i | 0.53 (0.52-0.54) | <0.001 |
| Insulin | 0.65 (0.64-0.65) | <0.001 |
| ACEI | 1.08 (1.07-1.08) | <0.001 |
| ARB | 1.09 (1.08-1.10) | <0.001 |
| Beta blockers | 0.99 (0.98-1.00) | <0.001 |
| Calcium channel blockers | 1.06 (1.06-1.07) | <0.001 |
| Thiazide diuretics | 1.06 (1.05-1.07) | <0.001 |
| Statin | 1.23 (1.22-1.24) | <0.001 |
| Other lipid-lowering agents | 1.14 (1.13-1.16) | <0.001 |
| Aspirin | 1.09 (1.08-1.10) | <0.001 |

Abbreviation: TZD, thiazolidinedione. SGLT-2i, sodium-glucose co-transporter-2 inhibitors. GLP-1 RA, glucagon-like peptide-1 agonist. DPP-4i, dipeptidyl peptidase-4 inhibitors. ACEI, angiotensin converting enzyme inhibitors. ARB, angiotensin receptor blockers.

All medication was analysed independently, compared to non-users. Adjustment for cirrhosis status (compensated cirrhosis, decompensated cirrhosis), age, gender, body mass index, type 2 diabetes mellitus duration, hypertension, dyslipidaemia, hepatitis virus infection, excessive alcohol intake, estimated glomerular filtration rate (eGFR), total bilirubin, total cholesterol, platelet, albumin, alanine aminotransferase and aspartate aminotransferase.

**Table S8.** Subgroup analysis on the percentage of patients achieving the HbA_1c_ target in type 2 diabetes patients without cirrhosis, with compensated cirrhosis and decompensated cirrhosis, stratifying by age and key comorbidities.

| Subgroups | Patients without cirrhosis (%) ^ | | | | | Patients with compensated cirrhosis (%) ^#^ | | | | | Patients with decompensated cirrhosis (%) ^@^ | | | | | *p* value ^$^ |
| --- | --- | --- | --- | --- | --- | --- | --- | --- | --- | --- | --- | --- | --- | --- | --- | --- |
|  | **2000-04**  **N=274,527** | **2005-09**  **N=414,676** | **2010-14**  **N=587,307** | **2015-19**  **N=765,409** | **2020-23**  **N=900,553** | **2000-04**  **N=7,917** | **2005-09**  **N=11,485** | **2010-14**  **N=16,756** | **2015-19**  **N=20,985** | **2020-23**  **N=25,719** | **2000-04**  **N=2,249** | **2005-09**  **N=3,800** | **2010-14**  **N=5,792** | **2015-19**  **N=6,898** | **2020-23**  **N=6,650** |  |
| Age |  |  |  |  |  |  |  |  |  |  |  |  |  |  |  |  |
| <65 years | 37.1 | 40.4 | 50.0 | 55.1 | 61.4 | 42.4 | 46.7 | 56.3 | 61.9 | 69.1 | 35.2 | 39.5 | 46.4 | 54.8 | 64.4 | <0.001 |
| ≥65 years | 43.0 | 51.4 | 64.1 | 71.8 | 76.5 | 50.7 | 59.3 | 73.2 | 80.1 | 84.3 | 44.9 | 51.8 | 61.9 | 70.4 | 77.0 | <0.001 |
| Hypertension |  |  |  |  |  |  |  |  |  |  |  |  |  |  |  |  |
| No | 39.2 | 44.3 | 53.4 | 58.7 | 65.9 | 49.4 | 54.5 | 64.2 | 70.8 | 75.5 | 47.8 | 51.0 | 54.3 | 60.4 | 69.1 | <0.001 |
| Yes | 40.4 | 45.7 | 55.9 | 61.5 | 66.7 | 49.9 | 57.1 | 69.2 | 73.7 | 78.1 | 50.8 | 51.5 | 55.7 | 63.3 | 68.9 | <0.001 |
| CKD |  |  |  |  |  |  |  |  |  |  |  |  |  |  |  |  |
| Moderate | 39.1 | 44.2 | 55.5 | 61.5 | 67.5 | 50.2 | 55.3 | 66.5 | 71.6 | 76.9 | 46.2 | 46.4 | 52.6 | 61.2 | 66.3 | <0.001 |
| Severe | 41.8 | 48.0 | 55.4 | 59.7 | 63.5 | 49.4 | 58.2 | 71.0 | 75.7 | 79.1 | 53.0 | 54.6 | 57.8 | 64.4 | 70.9 | <0.001 |
| MACE |  |  |  |  |  |  |  |  |  |  |  |  |  |  |  |  |
| No | 39.4 | 44.4 | 55.2 | 60.6 | 66.3 | 50.1 | 55.2 | 64.9 | 70.2 | 74.9 | 51.4 | 50.4 | 54.7 | 61.8 | 67.5 | <0.001 |
| Yes | 42.8 | 48.6 | 56.6 | 63.0 | 67.8 | 49.2 | 58.6 | 73.0 | 77.7 | 82.3 | 47.0 | 53.6 | 57.4 | 65.5 | 71.8 | <0.001 |

Abbreviation: CKD, chronic kidney dysfunction. MACE, major adverse cardiovascular events.

^ All P values <0.001 for linear trend in consecutive periods in patients without cirrhosis.

# All P values <0.001 for linear trend in consecutive periods in patients with compensated cirrhosis.

@ All P values <0.001 for linear trend in consecutive periods in patients with decompensated cirrhosis.

**Table S9.** Incidence of hypoglycaemia (per 100 person-years) among type 2 diabetes patients without cirrhosis, with compensated cirrhosis and decompensated cirrhosis over years.

| Group | Period | | | | |
| --- | --- | --- | --- | --- | --- |
|  | **2000-2004** | **2005-2009** | **2010-2014** | **2015-2019** | **2020-2023** |
| All patients | 1.79 (1.76, 1.83) | 0.91 (0.89, 0.94) | 0.68 (0.66, 0.70) | 0.65 (0.63, 0.67) | 0.55 (0.53, 0.57) |
| Non-cirrhosis | 1.73 (1.70, 1.76) | 0.77 (0.74, 0.80) | 0.50 (0.48, 0.51) | 0.48 (0.46, 0.50) | 0.36 (0.34, 0.38) |
| Compensated cirrhosis | 3.64 (3.34, 3.96) | 2.53 (2.24, 2.85) | 2.23 (2.02, 2.47) | 2.88 (2.62, 3.16) | 2.25 (2.02, 2.49) |
| Decompensated cirrhosis | 9.62 (8.38, 11.0) | 16.4 (14.9, 18.0) | 21.4 (19.9, 23.0) | 15.7 (14.5, 17.0) | 20.4 (18.7, 22.2) |

**References**

1. Cheng M, Tong Y, Kwok T, Cheng I, Chung A, Leung J, et al. Development journey of clinical data analysis and reporting system (CDARS) in hospital authority of Hong Kong. *Medinfo*. 2010;1468

2. Hong Kong 2016 population by-census - thematic report: ethnic minorities HKSAR: the Census and statistics department. <https://www.censtatd.gov.hk/en/EIndexbySubject.html?pcode=B1120100&scode=459>. Accessed March 8, 2026

3. Hospital authority strategic plan 2022-2027 HKSAR: the Hong Kong hospital authority. <https://www.ha.org.hk/haho/ho/ap/HA_StrategicPlan2022-2027_Eng_211216.pdf>. Accessed March 8, 2026

4. Fan JG, Kim SU, Wong VW. New trends on obesity and NAFLD in Asia. *J Hepatol*. Oct 2017;67(4):862-873. doi:10.1016/j.jhep.2017.06.003
